# Insights into Substrate Recognition by the Unusual Nitrating Enzyme RufO

**DOI:** 10.1021/acschembio.3c00328

**Published:** 2023-08-09

**Authors:** Benjamin D. Dratch, Kirklin L. McWhorter, Tamra C. Blue, Stacey K. Jones, Samantha M. Horwitz, Katherine M. Davis

**Affiliations:** Department of Chemistry, Emory University, 1515 Dickey Drive, Atlanta, Georgia 30322, United States

## Abstract

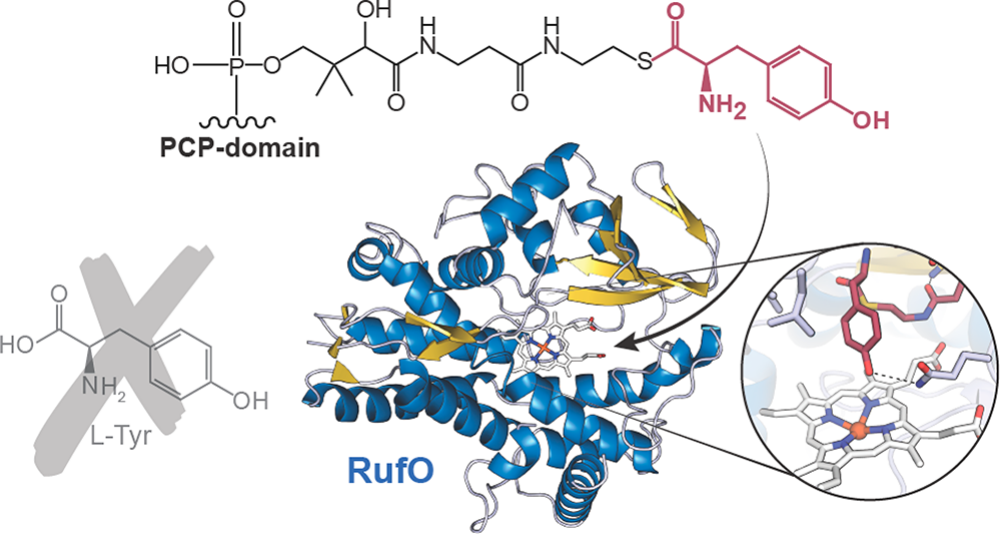

Nitration reactions are crucial for many industrial syntheses; however, current protocols lack site specificity and employ hazardous chemicals. The noncanonical cytochrome P450 enzymes RufO and TxtE catalyze the only known direct aromatic nitration reactions in nature, making them attractive model systems for the development of analogous biocatalytic and/or biomimetic reactions that proceed under mild conditions. While the associated mechanism has been well-characterized in TxtE, much less is known about RufO. Herein we present the first structure of RufO alongside a series of computational and biochemical studies investigating its unusual reactivity. We demonstrate that free l-tyrosine is not readily accepted as a substrate despite previous reports to the contrary. Instead, we propose that RufO natively modifies l-tyrosine tethered to the peptidyl carrier protein of a nonribosomal peptide synthetase encoded by the same biosynthetic gene cluster and present both docking and molecular dynamics simulations consistent with this hypothesis. Our results expand the scope of direct enzymatic nitration reactions and provide the first evidence for such a modification of a peptide synthetase-bound substrate. Both of these insights may aid in the downstream development of biocatalytic approaches to synthesize rufomycin analogues and related drug candidates.

Nitroaromatics are important molecular building blocks for the production of industrial commodities such as polymers, dyes, pesticides, explosives, and pharmaceuticals.^1−4^ Current synthetic routes to generate them, however, lack precise control over diastereospecificity and typically require highly acidic conditions.^5−7^ The discovery of two noncanonical cytochrome P450s (CYPs) from *Streptomyces*, TxtE and RufO, capable of installing nitro (NO_2_) groups during the biosynthesis of nonribosomal peptide natural products has renewed interest in developing more sustainable biocatalytic methods to produce such industrially relevant nitro compounds. In fact, these CYPs are the only enzymes discovered to date capable of efficiently catalyzing direct and regioselective nitration reactions, yet relatively little is known about the mechanism by which they achieve their novel reactivity.^8,9^

More specifically, TxtE is responsible for the production of 4-nitro-l-Trp as a precursor to the dipeptide phytotoxin thaxtomin A, while RufO supplies 3-nitro-l-Tyr for incorporation into the heptapeptide tuberculostatic agent rufomycin.^8,10,11^ The latter is a promising lead compound in the treatment of various multidrug-resistant mycobacterial infections and even some cancers.^12^ Previous studies suggest that these transformations diverge from the standard CYP catalytic cycle upon reaction of the ferric superoxo with nitric oxide (NO), likely afforded by an NO synthase directly preceding the CYPs within their respective biosynthetic gene clusters (BGCs), to yield a peroxynitrite adduct.^13^ Subsequent cleavage of the O–O bond would result in a ferryl heme and NO_2_ radical; however, the mechanism of nitration that follows remains unclear (Figure S1). Crystal structures of TxtE have been solved in complex with its substrate l-Trp, but the observed orientation of the indole does not favor nitration at the C4 position, making the basis for site-selective modification equally difficult to ascertain.

In an effort to gain insight into the basis for the remarkable regioselectivity of nitrating CYPs, we sought to structurally characterize RufO for comparison. RufO from *Streptomyces atratus* was therefore expressed and purified according to methods adapted from Tomita et al.^8^ before being crystallized via vapor diffusion approaches (see the Supporting Information for details). Herein we report the first crystallographic model of the enzyme, which we determined to a resolution of 1.87 Å (PDB entry 8SPC; Table S1). Generally speaking, RufO recapitulates the highly conserved CYP fold and comprises 12 α-helices (A–L) and five β-sheets (β1–4 and β6) (Figure 1A).^9,14^ Comparison with TxtE (PDB entry 4TPO) reveals minimal differences despite relatively low sequence homology (29%; Figure S2A). More specifically, the alignment of α-carbons in the peptide backbone yields a root-mean-square deviation (RMSD) of 3.26 Å over 365 atoms. The largest divergence can be found in the BC loop, which is commonly implicated in substrate binding by CYPs.^15^ Previous structures of TxtE depict ordering of this region (residues 59–90) upon complexation with l-Trp, resulting in the formation of two short α-helices (B′ and B″). The BC loop of RufO (residues 69–86), by contrast, displays minimal secondary structure, with helices B′ and B′′ almost undetectable. Not only does this comparatively abbreviated loop increase solvent access to the active site, but our data further indicate that it likely remains ordered in the absence of substrate (Figure 1B). Instead, we observe unusual destabilization of the F helix (residues 153–166), located at the distal face of the Cys-coordinated heme. Like the BC loop, the F helix and nearby structural motifs often play a role in substrate recognition within the CYP superfamily.^15^ The ensuing FG loop is highly disordered, as is seen in all structures of wild-type TxtE solved to date, and molecular dynamics (MD) studies of the l-Trp nitrating enzyme suggest that it may play a crucial role in orientating the substrate during catalysis.^16^

**Figure 1:**
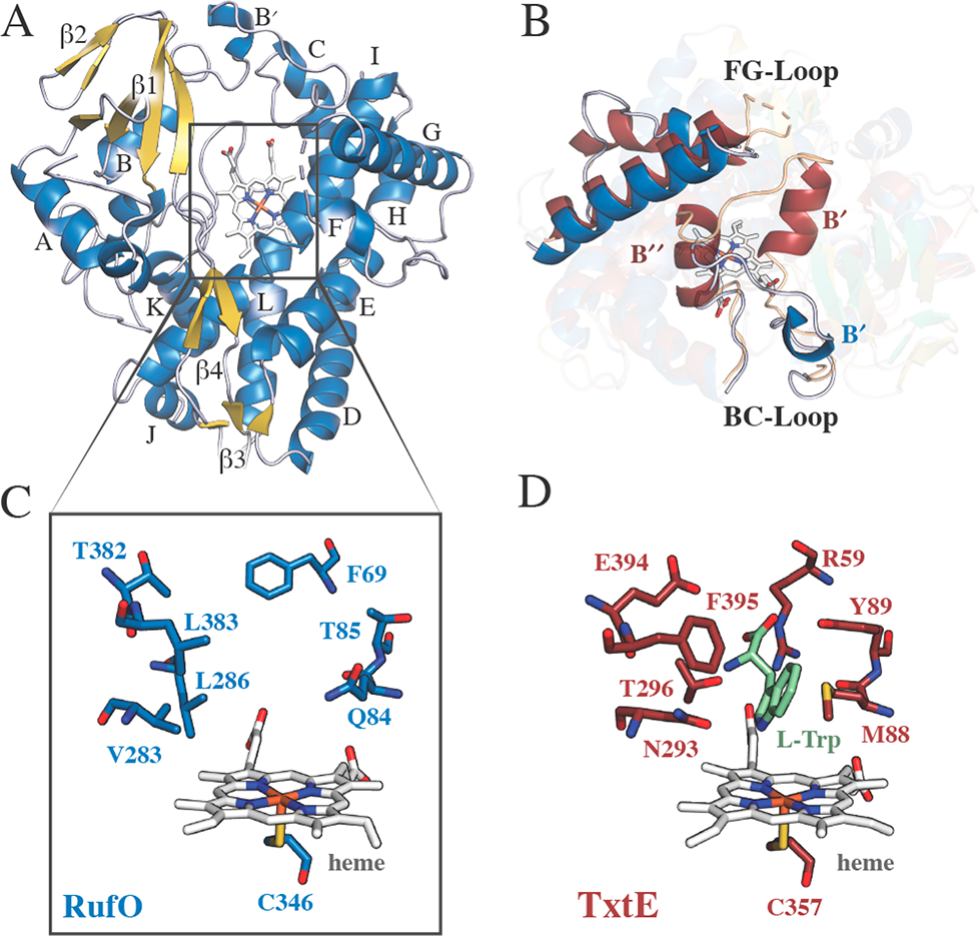
Structural comparison of RufO (PDB entry 8SPC) and l-Trp-bound TxtE (PDB entry 4TPO). (A) Overall fold of RufO with annotated α-helices and β-sheets. Note that β6 is too small to depict. (B) Loop regions facilitating access to the active site. (C) RufO and (D) TxtE active site residues surrounding the catalytic heme cofactor. RufO and TxtE α-helices/β-sheets/loops are shown in blue/gold/gray and red/green/tan, respectively. The heme cofactor is colored white and l-Trp in light green.

Beyond differences in large-scale structural features, the active site of RufO is substantially more hydrophobic than that of TxtE (Figure 1C,D). Many of the residues that form charged interactions directly with the amino and carboxylate moieties of l-Trp to promote substrate binding in TxtE (e.g., Arg59, Asn293, and Thr296) are replaced by nonpolar amino acids (e.g., Phe69, Val283, and Leu286) in RufO.^5,13,17^ An exception to this observation is the hydrophilic residue Gln84 from RufO. This residue occupies the same position as Met88 in TxtE, which is thought to work alongside Phe395 to bind the indole moiety of l-Trp.^5^ In addition to changes in polarity, residues lining the substrate binding pocket of RufO are generally less bulky than those in TxtE (Figure 1C,D), thereby increasing the volume of the active site by approximately 25% (Figure S2B). The physiological relevance of this altered binding pocket is unclear, given that the proposed free amino acid substrates are of a comparable size. Unfortunately, we were unable to obtain structures of RufO complexed with l-Tyr or 3-nitro-l-Tyr that might help to rationalize these differences, despite numerous attempts.

We therefore turned to UV–vis absorption spectroscopy to further investigate features of the RufO reactant complex. Reduction of the ferric enzyme resulted in a blue shift of the Soret peak from ∼421 to ∼411 nm. Additionally, we identified two Q bands at 543 and 570 nm in the spectrum of ferric RufO that coalesce to a single peak located at 547 nm upon reduction (Figure 2A). Given the potential for short-lived intermediates, we employed stopped-flow methods to study complexation with the cosubstrates O_2_ and NO. Rapid mixing of ferrous RufO with O_2_ resulted in the formation of a new species within the dead time of the instrument that is characterized by a Soret peak at ∼425 nm and a Q band at 561 nm (Figures 2B and S3). Such features are consistent with previously reported spectra of the short-lived ferric superoxo intermediate in TxtE and other CYPs.^13^ Formation of a putative NO-bound complex, by contrast, occurred within ∼400 ms and yielded peaks at 440 and 561 nm (Figures 2B and S4). This red shift in the Soret peak upon interaction of RufO with NO is similar to, but slightly more significant (∼5 nm) than that observed upon NO binding to TxtE.^13^ Nonetheless, the spectral features and kinetics of cosubstrate binding are remarkably consistent between the two enzymes, supporting the hypothesis that peroxynitrite formation in RufO likely occurs via the same mechanistic pathway as in TxtE.

**Figure 2:**
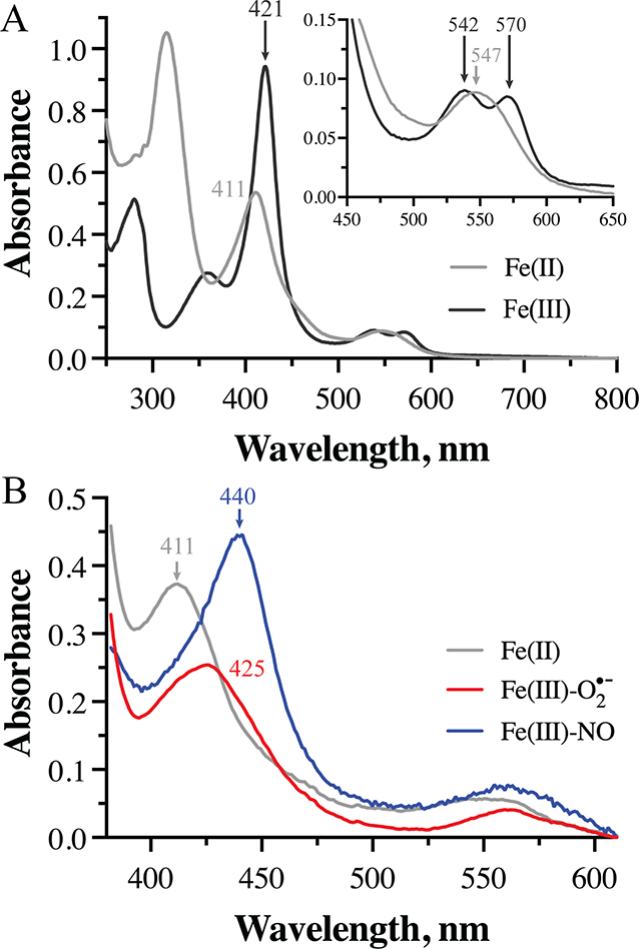
(A) UV–vis absorption spectra of substrate-free RufO in the ferric (black) and ferrous (gray) states. Q bands are magnified in the inset. (B) Spectral components representing the ferric superoxo (red) and NO-bound (blue) states of RufO determined via singular value decomposition and global fitting of stopped-flow data.

We were consequently quite surprised when our attempts to reproduce a blue shift of the Soret peak, reported previously upon incubation of ferric RufO with l-Tyr, were unsuccessful.^8^ Such a shift is typically indicative of substrate binding and displacement of the axial water ligand from the heme cofactor in CYPs. However, the spectrum of RufO remained unchanged following the addition of up to 20 equiv of l-Tyr dissolved in water (Figure 3A). We were only able to reproduce a blue shift to ∼370 nm when Tyr was dissolved in 1 M HCl. Unfortunately, the same 50 nm blue shift is also observed with HCl alone, suggesting that the change is due to inclusion of HCl rather than binding of the free amino acid reported previously.^8^ To further assess the ability of RufO to accept free l-Tyr as a substrate, we performed activity assays under a series of well-established conditions that either utilized dithionite for single turnover or included the ferredoxin and ferredoxin NADP^+^-reductase reducing system (see the Supporting Information for more details).^8,9,13,16,18^ All reactions were quenched with HCl for subsequent analysis via direct injection or HPLC-coupled mass spectrometry (LC-MS) (Figures 3B and S5–S7). Extracted ion chromatograms were monitored for a mass-to-charge ratio (*m*/*z*) around 227.06, corresponding to 3-nitro-l-Tyr, but no detectable peaks were observed that correlate to putative product formation, in agreement with our UV–vis data.^8,9,13^

**Figure 3:**
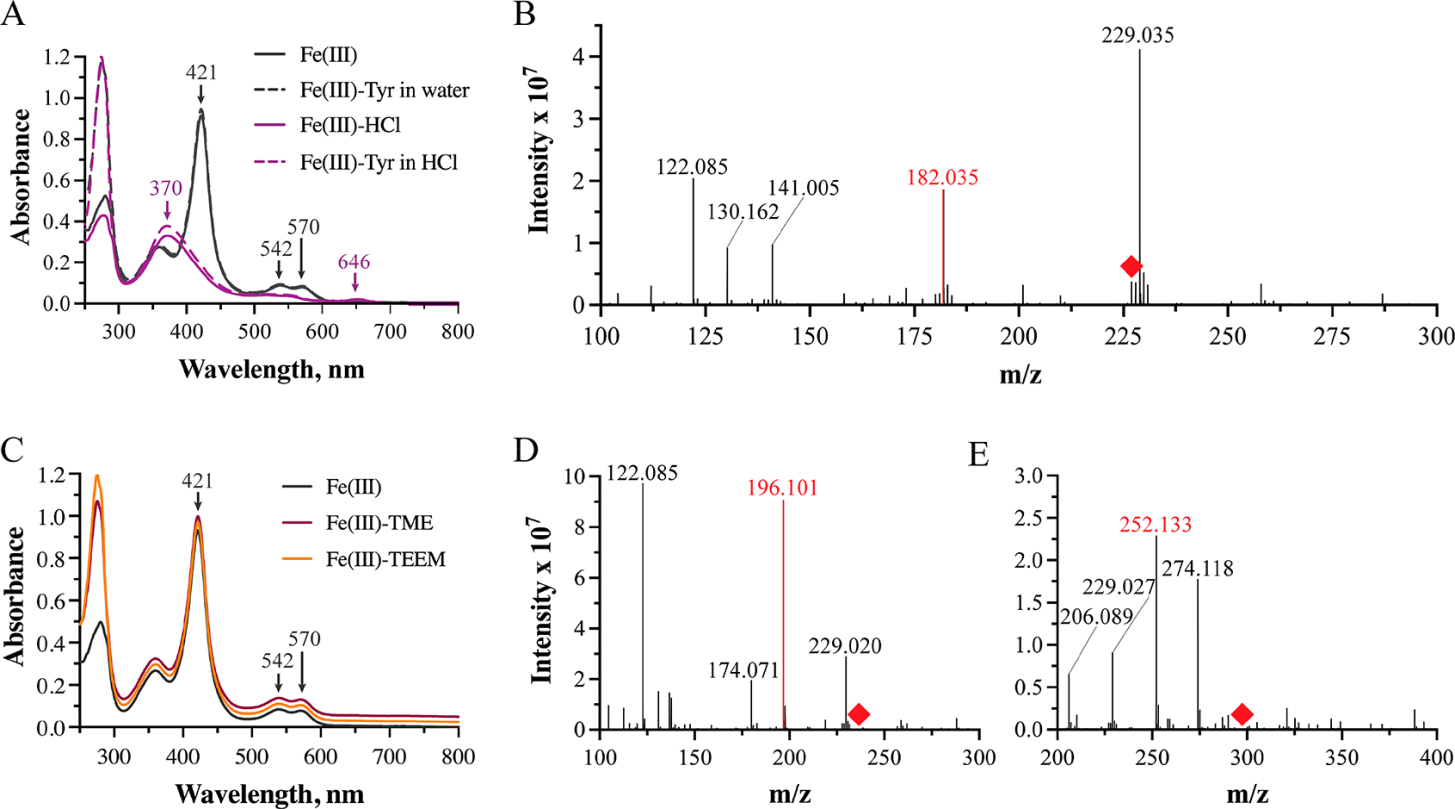
Absorption spectra and activity assays demonstrating the lack of significant l-Tyr binding to RufO. (A) Soret peaks and Q bands are unaltered by l-Tyr binding (black) and only shift following the addition of 1 M HCl (purple). (B) Direct-injection mass spectra of RufO reactions with l-Tyr (red peak). The red diamond indicates where 3-nitro-l-Tyr is expected (*m*/*z* [M + H]^+^ = 227.032). (C) Absorption spectra of RufO bound to l-Tyr analogues selected to mimic the amino acid bound to the phosphopantetheinyl arm of the PCP domain. Spectral traces have been offset for clarity. (D, E) Corresponding mass spectra from direct injection assessing reactivity with (D) TME or (E) TEEM. Red peaks correspond to these small molecules, while red diamonds indicate where nitrated products are expected (TME-NO_2_: *m*/*z* [M + H]^+^ = 242.082; TEEM-NO_2_: *m*/*z* [M + H]^+^ = 298.109).

Altogether, the aforementioned results prompted re-evaluation of RufO’s predicted function with particular emphasis on substrate identity. Both RufO and TxtE are produced by BGCs that also encode nonribosomal peptide synthetases (NRPSs). These large, multimodular enzymes recognize, activate, and sequentially link small-molecule substrates—commonly proteinogenic or modified amino acids—to generate complex natural products.^8,19,20^ The *txt* BGC, for example, encodes two single-module NRPSs, TxtA and TxtB, which bind l-Phe and 4-nitro-l-Trp, respectively, to produce the dipeptide core of thaxtomin A.^19,20^ The *ruf* BGC, by contrast, encodes a seven-module NRPS, RufT, the third module of which was previously hypothesized to incorporate 3-nitro-l-Tyr within the polypeptide scaffold of rufomycin following modification of the free amino acid by RufO.^8^ Our results run counter to this interpretation, as we see no evidence for significant RufO reactivity with free l-Tyr. Instead, we propose that RufO modifies the NRPS-bound amino acid during peptide assembly.

To interrogate this claim, we first performed a sequence analysis of conserved NRPS binding motifs. Substrate incorporation studies have identified an eight-residue core sequence that dictates the specificity of NRPS adenylation (A) domains.^21,22^ Using the prototypical A domain from GrsA as a reference, we identified the composition of this motif from the modules of RufT and TxtB, respectively.^23^ Comparison of the eight-residue core from the third RufT A domain reveals high similarity (≥75% sequence identity) to 10 of the 22 A domains identified by the NRPS substrate predictor database as l-Tyr binding (Table S2).^24^ The TxtB specificity motif is much more distinct, with most known l-Trp-binding A domains ≤50% identical (Table S3). This lack of sequence conservation in TxtB likely arises due to tailoring of the substrate binding pocket to preferentially accommodate 4-nitro-l-Trp over l-Trp. By contrast, the similarity of the relevant RufT A domain core sequence to known l-Tyr binding motifs is more consistent with incorporation of the unmodified amino acid. We hypothesize that a certain degree of promiscuity remains inherent to substrate recognition by the relevant RufT A domain, as previous studies report rufomycin production by Δ*rufO* mutants is restored upon the inclusion of 3-nitro-l-Tyr in the growth medium.^8,25^

It is not unusual for substrates selected by an A domain to be modified by tailoring enzymes, including many CYPs, following covalent attachment to the phosphopantetheinyl arm of the peptidyl carrier protein (PCP) domain.^26,27^ Because the substrate is esterified under these conditions, we first sought to repeat activity assays with the l-Tyr methyl ester (TME). To interrogate the possibility that nitration occurs after peptide bond formation, we also attempted assays with an N-acetyl l-Tyr ethyl ester (TEEM). In both cases, mass spectra yielded no peaks corresponding to a nitrated product, nor did incubation with the l-Tyr analogues produce a change in the postion of the Soret peak that could indicate binding to the active site (Figures 3C–E and S8–S10). It seems likely that these small molecules were insufficient to mimic interactions with the phosphopantetheinyl arm or other features of the PCP domain that may be required to stably orient the substrate in the active site.

Due to experimental challenges associated with generating PCP-bound l-Tyr, we pursued molecular docking studies to predict its binding mode to RufO. As no crystal structures exist for any portion of RufT, a model of the third PCP domain (residues 3029–3104), predicted to incorporate l-Tyr, was first generated using AlphaFold.^28^ Missing residues of RufO were also built *in silico* to obtain a complete structure of the CYP (see the Supporting Information for details). The resultant docking model of the complex depicts the PCP domain directly over the solvent-exposed active site of RufO (Figure 4A). Furthermore, the hydroxyl group of Ser36, which ultimately hosts the phosphopantetheine moiety, faces directly toward the heme cofactor. Such a conformation would likely position a PCP-tethered l-Tyr atom within the active site pocket. We subsequently appended the phosphopantetheinyl arm to the PCP domain *in silico* and again attempted molecular docking to simulate this binding mode. Unfortunately, the modeled conformation of the FG loop sterically occluded the placement within the active site. As this loop is a dynamic structure, we propose that it plays a gatekeeping role, controlling access to the active site; however, such behavior is difficult to capture computationally.

**Figure 4:**
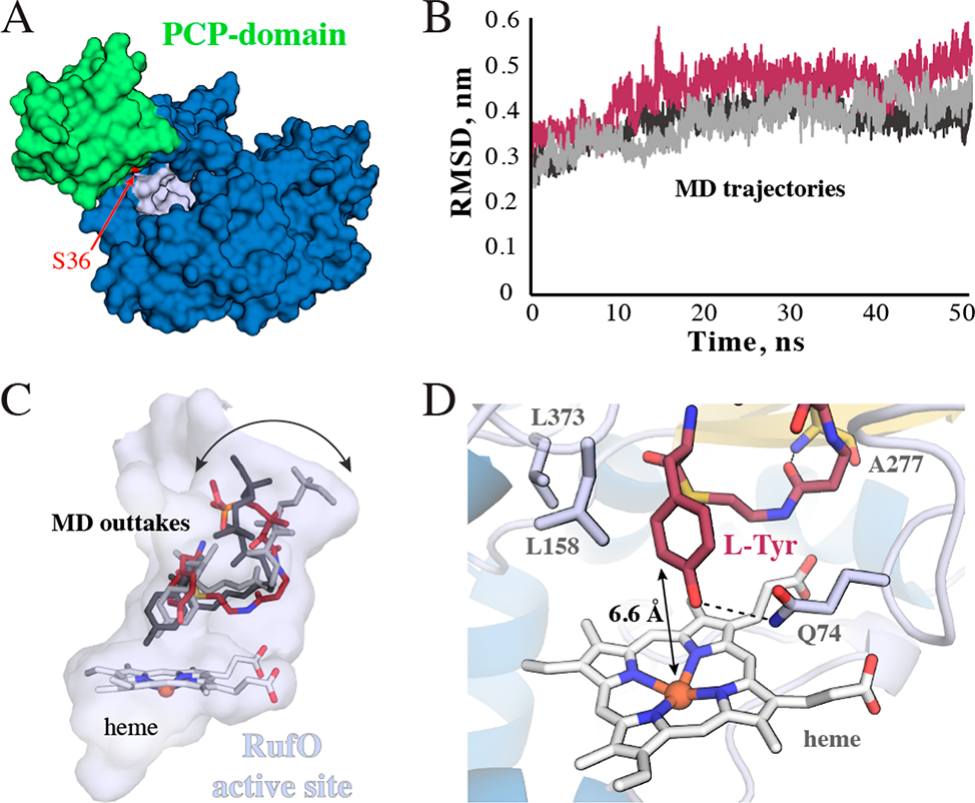
Computational prediction of the RufO substrate complex. (A) Surface model of the apo PCP domain bound to RufO. We highlight the serine that is ultimately phosphopantetheinylated in red. (B) Time-evolution RMSD plots of three independent 50 ns MD simulations of RufO in complex with the docked phosphopantetheinyl arm covalently linked to l-Tyr. (C) Snapshots of the three trajectories bound in the RufO active site pocket. Variation is primarily observed where the arm would attach to the PCP domain. (D) Representative configuration of the PCP-bound l-Tyr primed for the nitration reaction. The distance between the Fe ion and C3 is highlighted, as are key hydrogen bonds and hydrophobic contacts. See Figure S14 for a more complete interaction diagram. Secondary structure elements are colored as in Figure 1.

Thus, to predict interactions with the phosphopantetheine moiety, we docked the holo PCP domain to the incomplete crystallographic model of RufO. With much of the FG loop missing due to disorder, the phosphopantetheiynlphosphopantetheinyl arm was able to extend into the active site toward the heme cofactor. The physiological relevance and stability of this complex were subsequently evaluated after rebuilding the FG loop and covalently appending l-Tyr *in silico* via a series of MD simulations. Note that the putative PCP-bound substrate was truncated at the β-carbon of Ser36 to reduce computational cost. Intriguingly, the FG and BC loops were displaced from the active site opening over the course of these simulations, suggesting that while they appear to interact with the apo PCP domain (Figure S11), they may not directly associate with the phosphopantetheine moiety.

The l-Tyr-tethered phosphopantetheinyl arm, in contrast, remains stably positioned in the active site, as assessed by the time evolution of average RMSD values (Figure 4B). Furthermore, its configuration is remarkably consistent across all three trajectories, with the largest changes occurring near the entrance to the active site, likely due to the greater degree of freedom associated with the removal of the PCP domain (Figure 4C). A hydrogen-bonding interaction with the backbone of Ala277 is consistently observed, thereby restricting heterogeneity closer to the metallocofactor (Figure 4D). The hydrophobic nature of the active site also appears to play a crucial role in facilitating placement, making numerous contacts with the putative substrate (Figure S12). In particular, Leu158 and Leu373 form a hydrophobic pocket to accommodate the phenyl group of the l-Tyr moiety (Figure 4D). The exact orientation of l-Tyr, however, varies between the different simulations. The hydroxyl group either interacts with the heme Fe or a nearby glutamine side chain (Figure 4C,D). Excitingly, hydrogen bond formation with Gln74 places the site of nitration, C3, within 6.6 Å of the metal center in an orientation seemingly ideal for modification.

In conclusion, we report the first crystal structure of RufO. Careful analysis of the presented model reveals subtle but key differences between the enzyme and its l-Trp-nitrating counterpart, including a more open, hydrophobic active site, as well as changes to lid loops that are commonly implicated in CYP substrate binding. While spectroscopic characterization of the enzyme is consistent with the hypothesis that RufO performs a nitration reaction similar to that of TxtE via the formation of a peroxynitrite intermediate, we were unable to obtain any evidence to corroborate its previously reported reactivity with free l-Tyr. Instead, we propose that RufO modifies l-Tyr bound to the PCP domain associated with the third RufT module. Docking models support this revised order of peptide assembly and provide unique insights into the basis for substrate recognition by RufO. Although biochemical validation is still required, our results hint at unexpected versatility within nitrating CYPs and provide crucial insights into the biosynthesis of important lead compounds relevant for the treatment of drug-resistant mycobacterial infections.

## Supporting Information Available

Detailed description of materials and methods, MS data for enzymatic assays, crystallographic and refinement statistics, A-domain sequence analysis, stopped-flow data, docking simulations, and other supplemental figures (PDF)
