# Supplementary material for: Insights into Substrate Recognition by the Unusual Nitrating Enzyme RufO
Source: ACS Chem Biol. 2023 Aug 9;18(8):1713–8. doi: 10.1021/acschembio.3c00328 (PMC10442852; doi:10.1021/acschembio.3c00328)
Supplement: Supplementary file 1 — cb3c00328_si_001.pdf [file cb3c00328_si_001.pdf]

Supporting Information

Insights into substrate recognition by the unusual nitrating enzyme RufO

Benjamin D. Dratch, Kirklin L. McWhorter, Tamra C. Blue[α], Stacey K. Jones[α], Samantha M. Horwitz, Katherine M. Davis*

Department of Chemistry, Emory University, Atlanta, GA 30322, USA

*Correspondence: katherine.davis@emory.edu

[α]T.B. and S.J. contributed equally to this paper

Table of Contents

Materials and Methods 2
Supplementary Tables 7
Supplementary Figures 10
Supplementary References 22

Materials and Methods

Materials

Escherichia coli strains DH5α and BL21(DE3) were purchased from New England Biolabs (Ipswich, MA). 5-aminolevulinic acid (ALA) was purchased from Santa Cruz Biotechnology (Dallas, TX). L-Tyr was purchased from (Hampton, NH). 3-nitro-L-Tyr and DEA NONOate were purchased from Cayman Chemicals (Ann Arbor, MI). L-Tyr ethyl ester, N-acetyl L-Tyr ethyl ester monohydrate, ferredoxin, ferredoxin-$NADP^+$ reductase, and NADPH were purchased from Sigma Aldrich (St. Louis, MO). All other reagents used were of the highest purity commercially available.

Enzyme expression and purification

The synthesis and cloning of the gene encoding *rufO* from *Streptomyces atratus* into a pET28a(+) plasmid carrying a 6xHis N-terminal tag was performed by Twist Bioscience (San Francisco, CA). Expression and purification procedures for RufO (Unitprot: A0A224AU14) were adapted from previously determined methods described by Tomita *et al.*[1] In short, *E. coli* strain BL21(DE3) cells were transformed with the pET28a(+)/*rufO* plasmid using the heat shock method and incubated overnight on lysogeny broth (LB) agar plates containing 50 µg/mL kanamycin. We subsequently inoculated 150 mL of LB (25 g/L) containing 50 µg/mL kanamycin with a single colony of *E. coli* BL21 cells carrying the pET28a(+)/*rufO* plasmid. This starter culture was grown at 37 °C for 18 hours before subculturing 10 mL per 1 L of LB with 50 µg/mL kanamycin, 0.6 mM ALA and 0.15 mM $FeSO_4$. Cells were then incubated at 37 °C and shaken at 240 rpm until the cultures reached an OD_{600} = 0.6, after which IPTG was added to a final concentration of 0.1 mM. Following induction, cells were further incubated at 18 °C and 240 rpm for 20 hours.[2] Cells were harvested via centrifugation at 9,000 x *g* for 15 minutes then flash frozen in liquid nitrogen for storage at -80 °C.

All subsequent purification steps were performed at 4 °C to ensure protein stability. 4 mL of buffer A (20 mM NaPi, pH 8.0, 200 mM NaCl, and 5% glycerol) was added per g of cell paste, supplemented with 1X protease inhibitor cocktail, 1 mM phenylmethylsulfonyl fluoride (PMSF), 10 µg/mL DNase, and 1 mg/mL lysozyme. The resuspended cells were then lysed via sonication (10 s on, 10 s off for 45 minutes total at 50% amplitude). The resulting cell-free extract was collected by centrifugation at 32,000 x *g* for 30 min and loaded onto HisPur™ Ni-NTA resin equilibrated with 10 column volumes (CV) of buffer A. The column was then washed with 10 CVs of 10 mM imidazole in buffer A before elution at 40 mM imidazole in buffer A. The fraction containing RufO was concentrated using Thermo Fisher Scientific Pierce™ Protein Concentrators having a 30-kDa molecular weight cutoff and exchanged into buffer B (20 mM HEPES, pH 8.0, 400 mM NaCl, and 5% glycerol) using a Cytiva PD-10 column (Marlborough, MA) for further purification via size-exclusion. In order to obtain sufficient purity for crystallization, the enzyme was passed through a Cytiva HiLoad 16/600 Superdex 200 size exclusion column preequilibrated in buffer B and eluted using an isocratic gradient at 0.5 mL/min. Elution of RufO was monitored via absorbance at 280 nm and analyzed via SDS-PAGE. The purest fractions were pooled and concentrated to 25.4 mg/mL using Amicon Ultra filters with a 10-kDa molecular weight cutoff. Protein concentration was determined using a Thermo Fisher Scientific NanoDrop One^C (Waltham, MA) with an extinction coefficient of $\varepsilon_{280} = 29.45\ mM^{-1}\ cm^{-1}$ estimated by Expasy ProtParam.[3] Samples were then buffer-exchanged into 20 mM HEPES, pH 8.0, 20 mM NaCl, 0.1 mM DTT, and 5% glycerol, before being stored at -80 °C.

Crystallization of holo-RufO

The N-terminally 6xHis-tagged enzyme was first exchanged from the storage conditions above into a buffer comprised of 20 mM HEPES, pH 8.0, 20 mM NaCl, and 5% glycerol, and diluted to a final concentration of 13 mg/mL. Crystallization was performed via the hanging drop vapor diffusion method at room temperature, during which RufO was mixed 1:1 with a precipitant solution of 0.2 M $MgCl_2$, 0.1 M BIS-TRIS, pH 6.7, and 23% (w/v) PEG 3350 to generate a final drop volume of 2 μL. Red, cube-like crystals appeared within 3 days and were fully formed within 1 week. Upon looping, the crystals were soaked in a sub-stock of precipitant solution supplemented with 30% (v/v) ethylene glycol before flash freezing in liquid nitrogen.

X-ray data collection and processing

All data were indexed, integrated, and scaled using XDS before merging with AIMLESS.[4, 5] Model building and phasing/refinement were performed using Coot and Phenix, respectively.[6-8] The quality of the model was regularly assessed using Molprobity, and PDB_REDO was employed to check for model bias.[9, 10] Selected data processing and refinement statistics are presented in **Table S1**. Figures depicting the structure were generated with PyMol.[11] Solvent-accessible surface areas and volumes were calculated using the Computed Atlas of Surface Topography of proteins (CASTp) webserver (**Fig. S2**).[12]

X-ray diffraction data were collected at beamline 23-ID-B of the Advanced Photon Source at Argonne National Laboratory (Chicago, IL) using a Dectris Eiger X 16M detector. All crystals were maintained at 100 K to minimize x-ray induced damage. Preliminary data were collected using an inverse beam ($\Delta\phi$ = 0.2°, wedge = 15°) and an incident wavelength set to the absorption edge of Fe at 1.740 Å. Molecular replacement single anomalous dispersion (MR-SAD) was performed using the AutoRickShaw automatic structure solution pipeline to phase the data.[13, 14] As BLAST was unable to identify a PDB structure with sufficient sequence similarity with RufO to enable molecular replacement, an AlphaFold prediction of the apo-RufO structure was used as the search model.[15-17] A higher resolution X-ray dataset (1.87 Å) was subsequently obtained, for which associated images were collected sequentially ($\Delta\phi$ = 0.2°), with an incident wavelength of 0.9762 Å. The final solution was determined via isomorphous replacement. Note that the same R_{free} flags were maintained and extended from the lower resolution dataset to ensure unbiased refinement.[6] The resultant model contained electron density for a single molecule (residues 11-394, missing 167-180) and a heme cofactor in the asymmetric unit.

UV-vis absorption spectroscopy

UV-vis absorption spectra were recorded using an Agilent Cary 3500 spectrophotometer (Santa Clara, CA) with a final protein concentration of 20 μM in a solution of 20 mM HEPES, pH 8.0, 20 mM NaCl, and 5% glycerol. Ferrous RufO samples were prepared in a Coy Laboratory Products anaerobic chamber (Grass Lake, MI) containing an atmosphere of approximately 97% N_2 and 3% H_2. All solutions were purged of O_2 prior to use. RufO was reduced to the ferrous state by the addition of 1 mM dithionite and transferred to a sealed cuvette prior to measuring the absorption spectrum. Spectroscopic analysis revealed the enzyme remained reduced for several days using this preparation. The effect of L-Tyr on RufO absorption spectra was determined via titrating L-Tyr solubilized in either water or 1 M HCl. The effect of HCl alone was determined by titrating in an equivalent amount of 1 M HCl. Representative RufO spectra depicting the addition of 400 μM L-Tyr, 120 mM HCl, or both were chosen for Fig. 3A. Resulting spectra were corrected for dilution factor and absorbance at 800 nm. The effect of L-Tyr analogues on the absorption spectra of RufO was determined by the addition of 400 μM L-Tyr methyl ester (TME) or N-acetyl L-Tyr ethyl ester monohydrate (TEEM).

Stopped-flow spectroscopy

UV-vis absorption spectra of transient complexes were recorded using a two-syringe OLIS RSM 1000 stopped-flow spectrophotometer (Bogart, GA) equipped with a thermostated water bath. All experiments were carried out at 4 °C with a final protein concentration of 20 μM. Protein and substrate solutions were prepared in 20 mM HEPES, pH 8.0, 20 mM NaCl, and 5% glycerol inside an anerobic chamber, as previously described. To ensure the ferrous state was maintained prior to fast-mixing on the stopped-flow device, excess dithionite (30 mM) was added to 40 μM of ferric enzyme to generate the reduced state. The oxidation state was confirmed by UV-visible spectroscopy utilizing a sealed cuvette, similar to experiments described above. To remove oxygen from the stopped-flow lines, the system was equilibrated with 30 mM dithionite, before washing with anaerobic buffer. NO-binding to the ferrous enzyme was assessed by mixing with a solution of 800 μM DEA NONOate prepared in degassed buffer. Similarly, O_2-binding was assessed by mixing with buffer bubbled with O_2 for 20 s at 4 °C (~400 μM).[18] Time-resolved spectral changes were recorded using a DeSa rapid-scanning monochromator equipped with a 0.6 mm entrance slit and a grating of 400 lines per meter and 500 nm blaze wavelength. Single value decomposition and global analysis was performed using Olis GlobalWorks to generate representative spectra of the Fe(III)-$O_2^{\bullet-}$ and Fe(III)-NO traces (**Figs. S3 & S4**).

LC-MS analysis of RufO activity

Various reaction conditions were tested to ensure the lack of detectable product was not due to experimental setup, including permutations of substrate, DEA NONOate, peroxynitrite, ferredoxin, and ferredoxin-$NADP^+$ reductase in a buffer of 25 mM Tris-Cl, pH 8.0, and 5% glycerol. All reaction mixtures were first incubated with RufO and substrate for 30 min at 23 °C to allow for binding. To assess activity with small molecule mimics for the phosphopantetheinyl arm of a PCP-bound L-Tyr substrate, L-Tyr was replaced with TME or TEEM. Reactions were either set up under anaerobic or aerobic conditions. For anaerobic conditions, the reactions were prepared in an anaerobic chamber, as previously described, and dithionite was used to reduce the enzyme to the ferrous state. Anaerobic reactions were initiated by removing the samples from the glovebox, bubbling with air briefly to introduce O_2, and then inverted to mix. Aerobic reactions were prepared outside the glovebox, where NADPH, ferredoxin, and ferredoxin-$NADP^+$ reductase were utilized as a reducing system. Specific conditions for each activity assay can be found in **Figures S5-10**. Reactions were carried out for 2 hrs at 23 °C, after which 20 μL of 1 M HCl was applied to solubilize potential products and denature RufO. Reaction mixtures were subsequently passed through Millipore 0.5 mL 3-kDa molecular weight cutoff centrifugal filters, and product formation was assessed via electrospray ionization mass spectrometry (ESI-MS) operated in positive ion mode at the Georgia State University or Emory University Mass Spectrometry Facilities. Standards containing only L-Tyr, 3-nitro-L-Tyr, TME, or TEEM were analyzed with each mass spectrometer to ensure the proper m/z values for each molecule were analyzed in subsequent experiments.

For direct injection MS, we utilized a Waters Xevo G2-XS Mass Spectrometer (Milford, MA) at Georgia State University. Samples were diluted by 100x with water, then 5 μL aliquots were introduced into the ion source through an autosampler with a flow rate of 200 μL/min. The instrument operation parameters were optimized as follows: capillary voltage of 1000 V, sample cone voltage of 20 V, desolvation temperature of 350 °C, and a source temperature of 120 °C. Nitrogen was used as cone gas and desolvation gas at flow rates of 25 and 800 L/h, respectively. Resulting mass spectra were acquired over a scan range of 50-800 m/z. MassLynx 4.2 software was used for data acquisition and processing. Resulting extracted ion chromatograms (EICs) were analyzed using MATLAB.

For LC-MS, a Thermo- LTQ Orbitrap Velos Mass Spectrometer equipped with a Thermo Dionex Ultimate 3000 dual pump, DAD, and a Shimadzu SIL-20AC HT autosampler at Emory University was employed.

The instrument was controlled with Thermo Xcalibur and Chomeleon Xpress. Mass spectra were taken at a resolution of 60,000 for each of the spectra with the agc set at 50,000 and a maximum injection time of 100 ms. The source used a heated electrospray ionization probe at 3.0 kV with a source temperature of 350 °C. The sheath gas flow rate was set at 35 arbitrary units and the aux flow rate at 5 arbitrary units. The capillary temperature was set to 320 °C and the S lens Rf level set to 60%. A 10 µL injection volume per sample was used. The chromatography used a Zorbax RRHD Eclipse Plus C18 column at a 0.2 mL/min flow rate equilibrated in 98% solvent A (water) and 2% solvent B (acetonitrile). A multi-step gradient 2-60% of solvent B was applied from 0.1-10 min. Subsequently, a gradient of 60-95% of solvent B was applied from 10.01-13 min. The column was equilibrated for 5 min before subsequent sample injections**.** Resulting EICs were analyzed using Thermo Freestyle.

Sequence similarity analysis of NRPS substrate-binding pockets

The sequences of 1,547 A-domains were downloaded from the non-ribosomal peptide synthase substrate predictor database (NRPSsp). Among these, 22 distinct sequences were annotated as L-Tyr-binding domains and 15 as L-Trp-binding domains.[19] This subset of sequences, along with TxtB and the A-domain from the third module of RufT (UniProt: A0A224ANA9), were aligned to the A3-A6 phenylalanine activation domain sequence of GrsA via the Clustal method in Jalview.[20, 21] Using methods described by the NRPS prediction blast server, the 8 core amino acids lining the binding pocket of each A-domain were identified.[20]

In silico analysis of RufO binding to the third PCP-domain from RufT

As no crystal structures exist for any portion of RufT, a model of the PCP-domain (residues 3029-3104) was first generated using AlphaFold. The phosphopantetheinyl moiety of the PCP-domain was subsequently modeled onto Ser36 in Pymol, and the prosthetic group was optimized using the program's 'clean' command, which performs an energy minimization using the MMFF94 forcefield. Docking was then performed using the High Ambiguity Driven protein-protein DOCKing (HADDOCK) 2.4 server.[22, 23] All simulations were run with default parameters for protein-protein interactions, excluding the minimum percentage of relative solvent accessibility (RSA), which was defined to consider a residue as accessible at 5.0%. To generate a structure of the complete enzyme, residues corresponding to the unmodeled FG-loop in RufO (residues 166-181) were added using COOT. The final rigid body template upon inclusion of this loop was generated via a 30 ns molecular dynamics (MD) simulation in GROMACS (see below for details). The docked complex formed between the apo-PCP-domain and the complete CYP enzyme was used to define ambiguous interaction restraints (AIRs) for subsequent HADDOCK runs with the phosphopantetheinyl-bound PCP-domain and the incomplete crystallographic model of RufO. A model in which the phosphopantetheinyl arm was observed reaching for the heme cofactor in the active site of RufO was utilized for further analysis.

Molecular dynamics simulations of RufO

To ensure the docked conformation of the phosphopantetheinyl arm represented a stable configuration, MD simulations were performed. A structure of the docking model with just the phosphopantetheinyl arm was obtained by replacing RufT with a methyl group at the β-carbon of Ser36. [24, 25] The carboxyl group of L-Tyr was attached to the free thiol of phosphopantetheinyl arm using Avogadro, and the geometry minimized using steepest descent over 1000 steps with constraints placed on the hydroxyl O of L-Tyr, the phosphate P of phosphopantetheinyl moiety, and all surrounding amino acids. Parameters for the phosphopantetheinyl moiety were obtained using AmberTools20,[23] and atomic charges were derived using the AM1-BCC method in antechamber.[24] Protonation states of amino acid side chains were assessed using the H++ tool,[26-28] and the resulting topology and coordinate files were used to generate a protonated structure in AmberTools. To obtain the parameters for the high spin (S=2) heme of RufO, the Metal Center Parameter Builder python

extension (MCPB.py) was used.[29, 30] Bond and angle force constants in MCPB.py were derived using the B3LYP functional,[31, 32] the cc-pVTZ basis for the Fe, and cc-pVDZ for the remaining atoms using Gaussian 09.[33] The remainder of the protein was parameterized using the Amber99SB-ILDN forcefield.[34, 35] AMBER parameters were translated to GROMACS compatible topologies using acpype.[36]

In preparation for simulations carried out using GROMACS 2019.3[37] and the Amber99SB-ILDN forcefield, the phosphopantetheinyl-bound RufO complex was placed in the center of an octahedral box (~10 Å from each hexagonal face), and subsequently solvated using the TIP3P model of water. Neutralization of the system was carried out by adding Na^+ counterions. The system was then subjected to 5000 steps of energy minimization using steepest descent, followed by controlled heating to 300 K at an NVT ensemble using the velocity rescaling thermostat over 500 ps.[38] The NPT ensemble was obtained using a Parrinello Rahman barostat set to 1 bar.[39] Note that the LINCS algorithm was used to constrain bonds involving hydrogens, and protein-ligand complex positions were restrained using a harmonic potential of 1000 kJ mol^{-1} nm^{-1} during the heating process.[40] Production MD runs were carried out over 50 ns each with a time step of 2 fs and a target pressure of 1 bar. Structure files from the resulting simulations were extracted for analysis using Chimera 2021-6-26.[41] RMSD analysis was carried out relative to the motion of the protein and considered non-hydrogen atoms from the entire phosphopantetheinyl arm.

Supplementary Tables

Table S1. Crystallographic data processing and refinement statistics for holo-RufO.

**PDB ID**	**8SPC**
**Data Collection**[a]	
Space group	$P4_12_12$
Unit cell (Å)	a = 77.627 b = 77.627 c = 136.898<br>α = β = γ = 90
Wavelength (Å)	0.9762
Resolution range (Å)	39.34 - 1.871 (1.937 - 1.871)
Total observations	449980 (28463)
Total unique observations	35269 (3398)
I/σ_I	15.29 (1.14)
Completeness (%)	99.56 (96.32)
R_{merge}	0.09647 (1.469)
R_{pim}	0.02792 (0.5285)
Multiplicity	12.8 (8.4)
**Refinement Statistics**	
Resolution range (Å)	39.34 - 1.871
Reflections (total)	35201 (3349)
Reflections (test)	3197 (298)
Total atoms refined	3156
Solvent	218
R_{work} (R_{free})	0.1940 (0.2167)
RMSDs	
Bond lengths (Å)/angles (º)	0.008/0.84
Ramachandran plot	
Favored/allowed (%)	97.54/2.46
**Mean B values (Å^2)**	
Protein chains A/B	39.92
Heme	33.98
Solvent	44.98

[a]Values in parentheses refer to the high-resolution shell.

Table S2. Comparison of the relevant RufT substrate binding motif to known L-Tyr-binding A-domains.

UniProt Accession[a]	Position								Identity (%)
	183	184	187	226	253	255	281	289	
A0A224ANA9 (RufT module 3)[b]	**D**	**A**	**S**	**T**	**A**	**A**	**A**	**V**	-
A6P624	**D**	**A**	**S**	**T**	L	**A**	**A**	**V**	88
Q70AZ9	**D**	**A**	**S**	**T**	V	**A**	**A**	**V**	88
Q7WZ66	**D**	**A**	**S**	**T**	V	**A**	**A**	**V**	88
Q8KLL3	**D**	**A**	**S**	**T**	V	**A**	**A**	**V**	88
Q93N87	**D**	**A**	**S**	**T**	V	**A**	**A**	**V**	88
Q9K5M1	**D**	**A**	**S**	**T**	I	**A**	**A**	**V**	88
Q9RAH1	**D**	**A**	**S**	**T**	I	**A**	**A**	**V**	88
B9ZUK5	**D**	**A**	**S**	**T**	V	**A**	**A**	I	75
Q939Y2	**D**	**A**	A	**T**	L	**A**	**A**	**V**	75
Q0VZ70	**D**	G	**S**	**T**	I	T	**A**	**V**	63
O30409	**D**	**A**	L	**T**	T	G	E	**V**	50
O30980	**D**	G	T	I	T	**A**	E	**V**	36
O87704	**D**	G	T	L	T	**A**	E	**V**	36
P39846	**D**	G	T	I	T	**A**	E	**V**	36
P94459	**D**	G	T	I	T	**A**	E	**V**	36
Q6Q883	**D**	**A**	G	**T**	F	G	C	C	36
Q9R9J0	**D**	**A**	L	S	V	G	E	**V**	36
Q93I55	**D**	**A**	L	S	V	G	E	**V**	36
Q83VS0	**D**	**A**	P	F	G	R	G	T	25
A0JJU1	**D**	M	V	I	C	G	C	A	13
Q5ATG8	**D**	M	V	I	C	G	C	A	13
Q9I157	**D**	V	W	H	F	G	R	I	13

[a]Sequences and UniProt accession codes were obtained from NRPSps.
[b]Bold and colored amino acids are consistent with the module 3 A-domain of RufT.

Table S3. Comparison of the TxtB substrate binding motif to known L-Trp-binding A-domains.

UniProt Accession[a]	Position								Identity (%)
	233	234	237	276	305	307	332	340	
C9ZDC0 (TxtB)[b]	**D**	**G**	**W**	**T**	**V**	**V**	**A**	**V**	-
Q9F0D7[c]	**D**	**G**	**W**	**T**	**V**	**V**	**A**	**V**	100
Q8CJX2	**D**	**G**	**W**	A	**V**	A	S	**V**	63
A8M6W3	**D**	V	**W**	**T**	H	G	G	**V**	50
B7X8E8	**D**	A	**W**	**T**	**V**	T	G	**V**	50
Q9Z4X6	**D**	A	**W**	S	**V**	G	S	**V**	50
Q93N89	**D**	V	A	V	**V**	G	E	**V**	36
A1DN09	**D**	V	M	F	**V**	G	E	**V**	36
Q45R85	**D**	V	A	L	**V**	G	V	**V**	36
Q4WAW3	**D**	V	M	F	I	G	**A**	**V**	36
Q50E74	**D**	V	S	S	I	G	**A**	**V**	36
Q70LM4	**D**	V	S	S	I	G	C	**V**	25
Q70LM5	**D**	V	S	S	I	G	C	**V**	25
A8KNE2	**D**	M	I	I	C	G	C	A	13
B6F209	**D**	M	A	L	C	G	S	A	13
C9K4U2	**D**	M	A	L	C	G	S	A	13

[a]Sequences and UniProt accession codes were obtained from NRPSps.
[b]Bold and colored amino acids are consistent with TxtB.
[c]Sequence represents a homologue of TxtB from *S. acidiscabies*.

Supplementary Figures

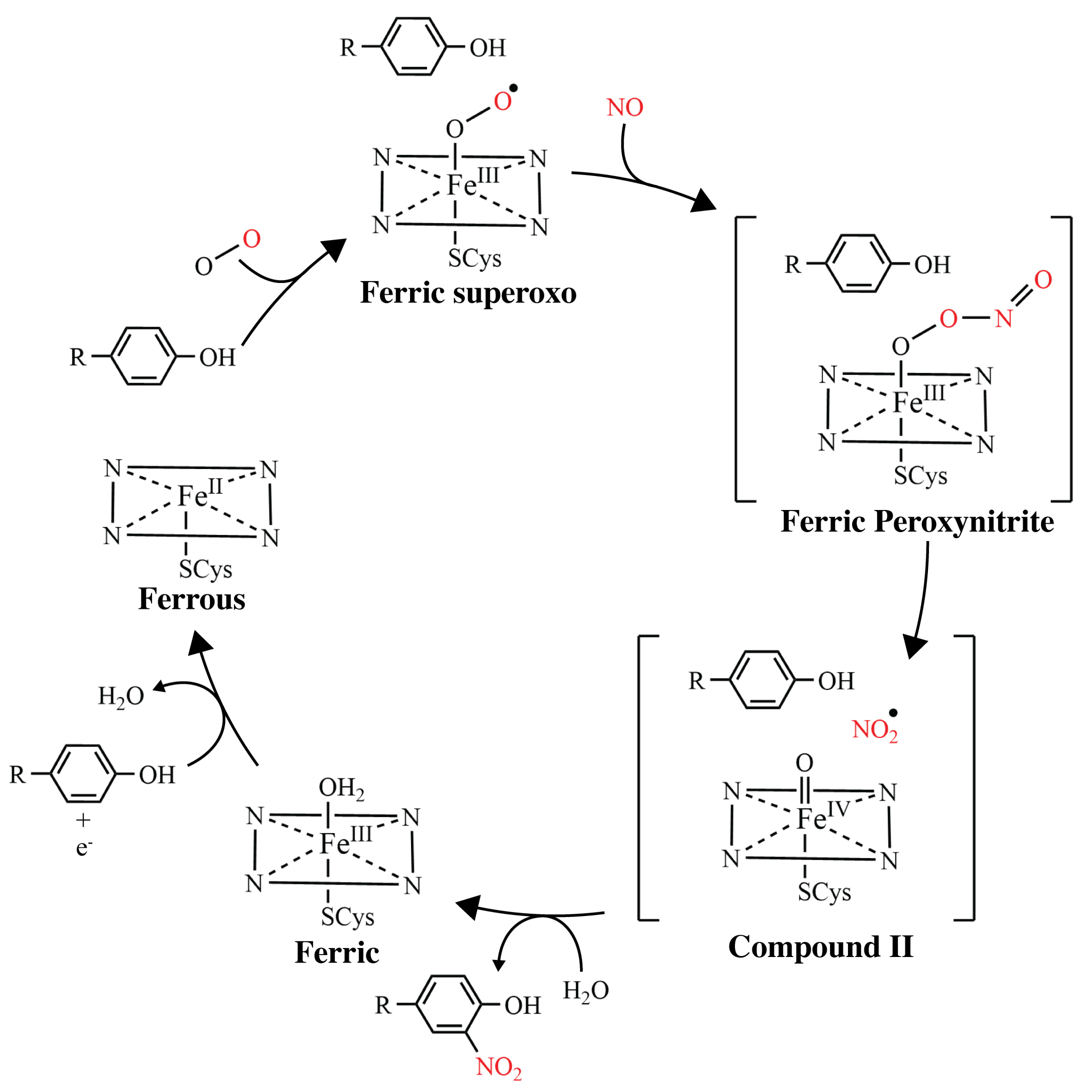

Figure S1. Hypothetical mechanism of RufO-catalyzed direct nitration. Intermediates are based on those predicted for TxtE (UniProt: C9ZDC6).[42] Atoms colored in red form the nitro-group of the 3-nitro-L-Tyr product.

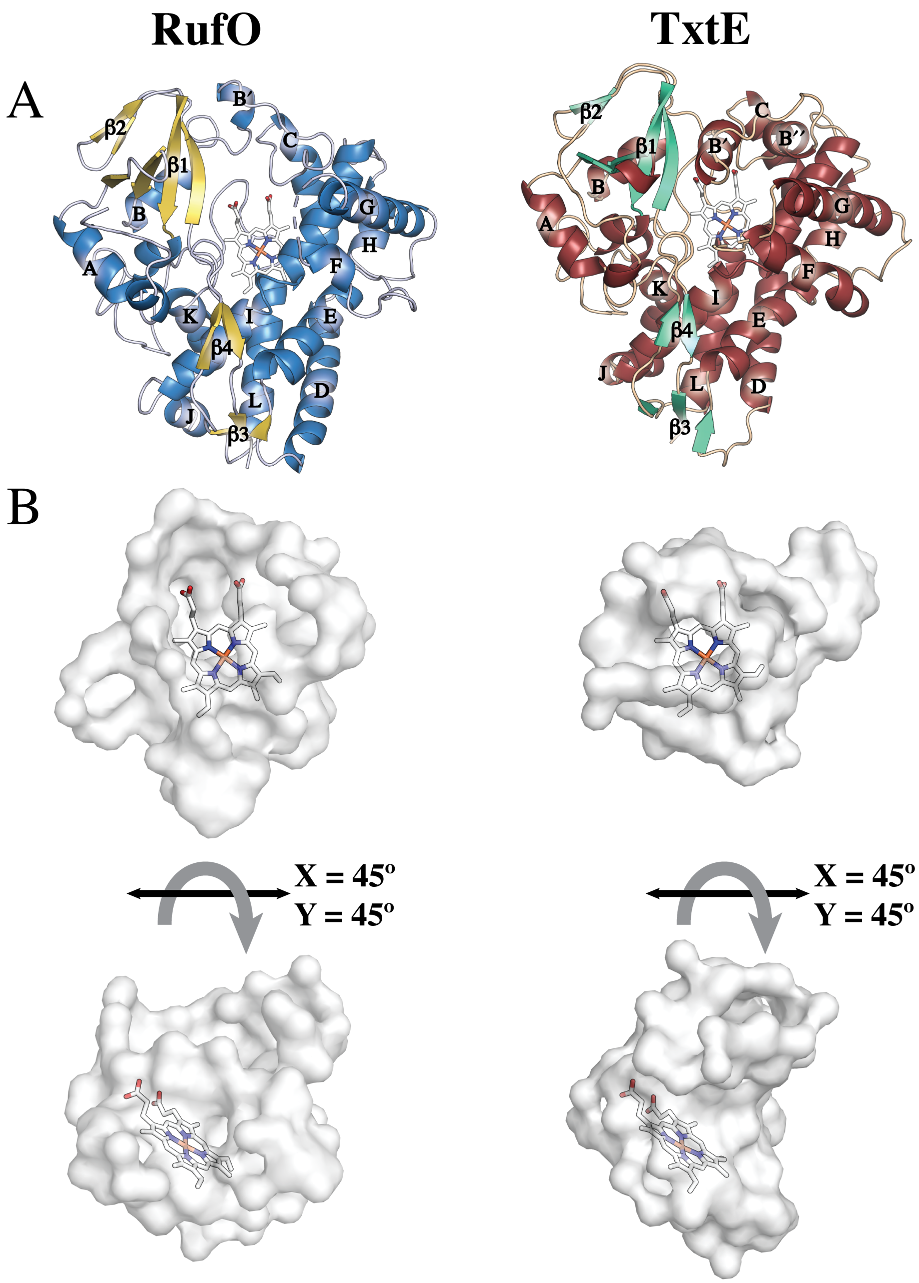

Figure S2. Structural comparison of RufO (PDB: 8SPC) and TxtE (PDB: 4TPO). (A) The overall fold of the two enzymes is nearly identical, apart from the B'' α-helix, which is only present in TxtE. Features are colored as in Fig. 1. (B) Visualization of the solvent-accessible active site pockets of RufO and TxtE generated by CASTp reveals a larger substrate binding pocket in RufO. The solvent-accessible areas and volumes calculated by CASTp were 1060 $Å^2$ and 830 $Å^3$ for RufO and 1080 $Å^2$ and 620 $Å^3$ for TxtE.

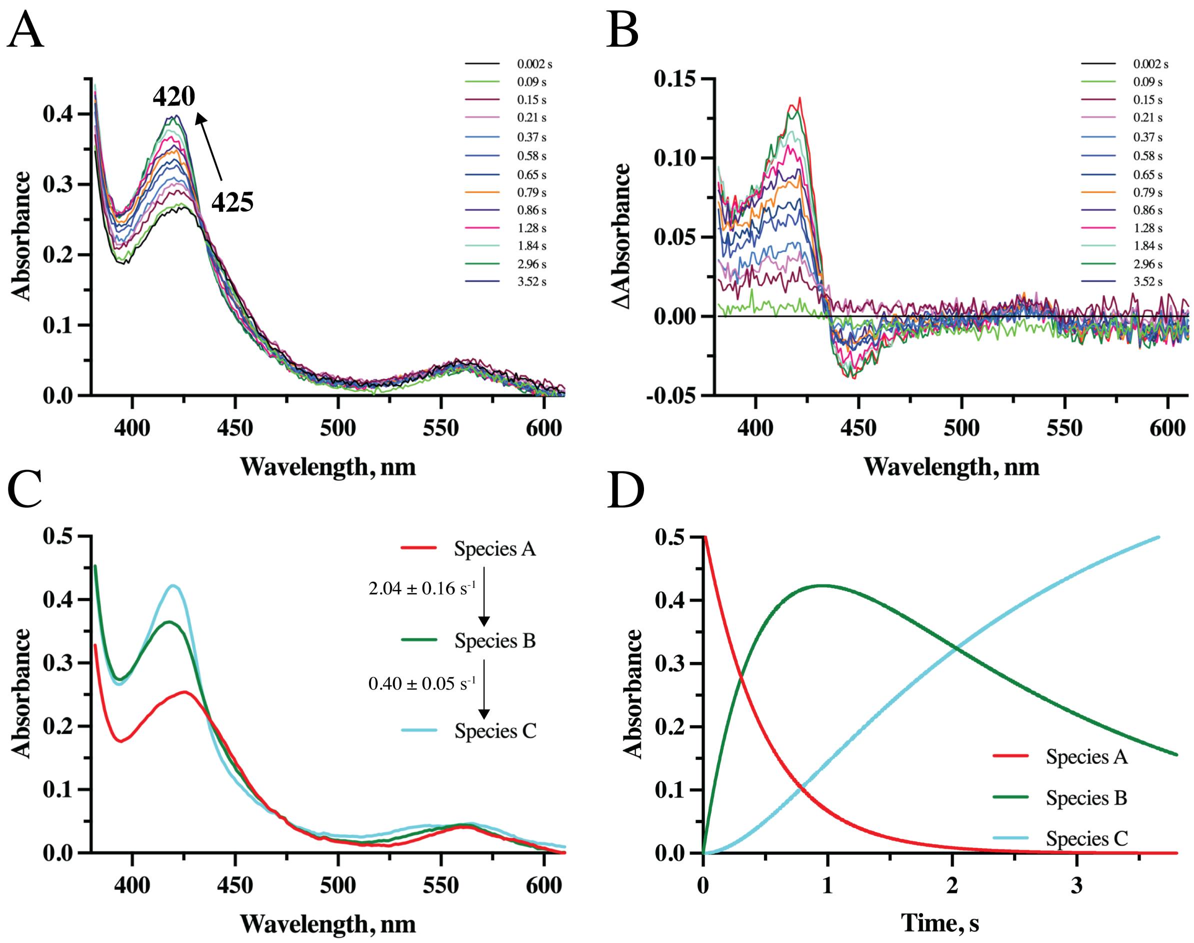

Figure S3. Stopped-flow data upon fast-mixing with O_2. (A) Absorption and (B) difference spectra show a peak at 425 nm, consistent with the formation of a ferric-superoxo species within the dead time of the instrument, which decays to the ferric resting state over ~3.9 s. SVD and global fitting analysis of the data yields (C) three distinct spectral components and (D) corresponding kinetic traces. Species A corresponds to the ferric-superoxo, while species C aligns with the ferric resting state of the enzyme. The spectrum of species B is consistent with a water-bound ferric species, as was observed for TxtE.[42]

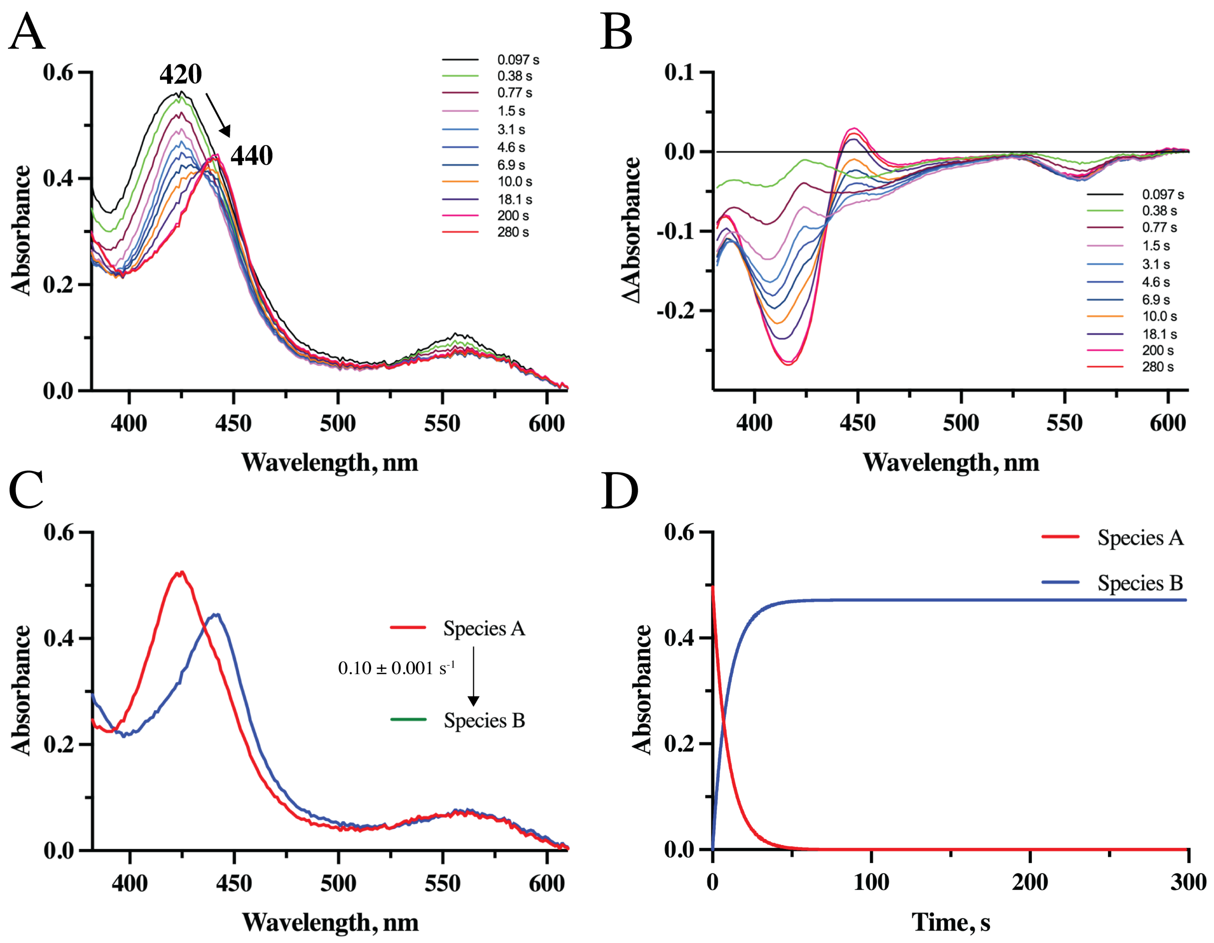

Figure S4. **Stopped-flow data depicting NO-binding.** (A) Absorption and (B) difference spectra captured over 300 s show a shift in the Soret from 420 nm to 440 nm, representing the formation of an Fe(III)-NO complex. SVD and global fitting analysis of the data yields (C) two distinct spectral components and (D) corresponding kinetic traces. Note that the NO-bound species dominates after 400 ms.

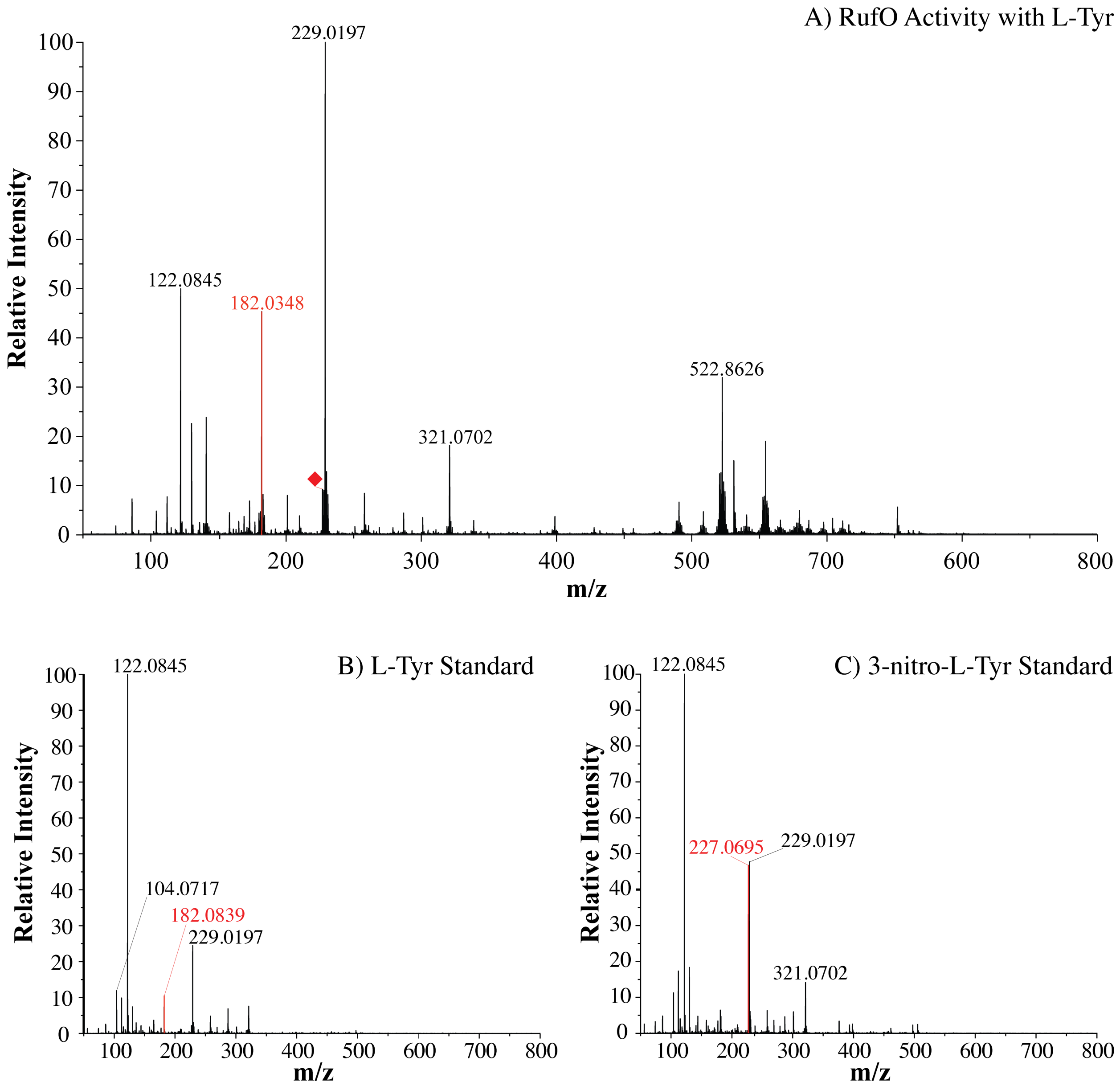

Figure S5. Direct injection MS analysis of a single turnover RufO reaction with L-Tyr. Full mass spectra from direct injection of (A) the reaction mixture, (B) 1 mM L-Tyr standard, and (C) 1 mM 3-nitro-L-Tyr standard. Red peaks highlight either L-Tyr (calculated m/z [m+H$^+$] = 182.032) or 3-nitro-L-Tyr (calculated m/z [m+H$^+$] = 227.032), while the red diamond indictes where product formation is expected. The enzymatic mixture was prepared under anaerobic conditions with 4 µM RufO, 1 mM L-Tyr, 1 mM NADH, and 1 mM DEA NONOate in 25 mM Tris-Cl, pH 8.0, and 5% glycerol. Small molecule standards were prepared in 25 mM Tris-Cl, pH 8.0, and 5% glycerol.

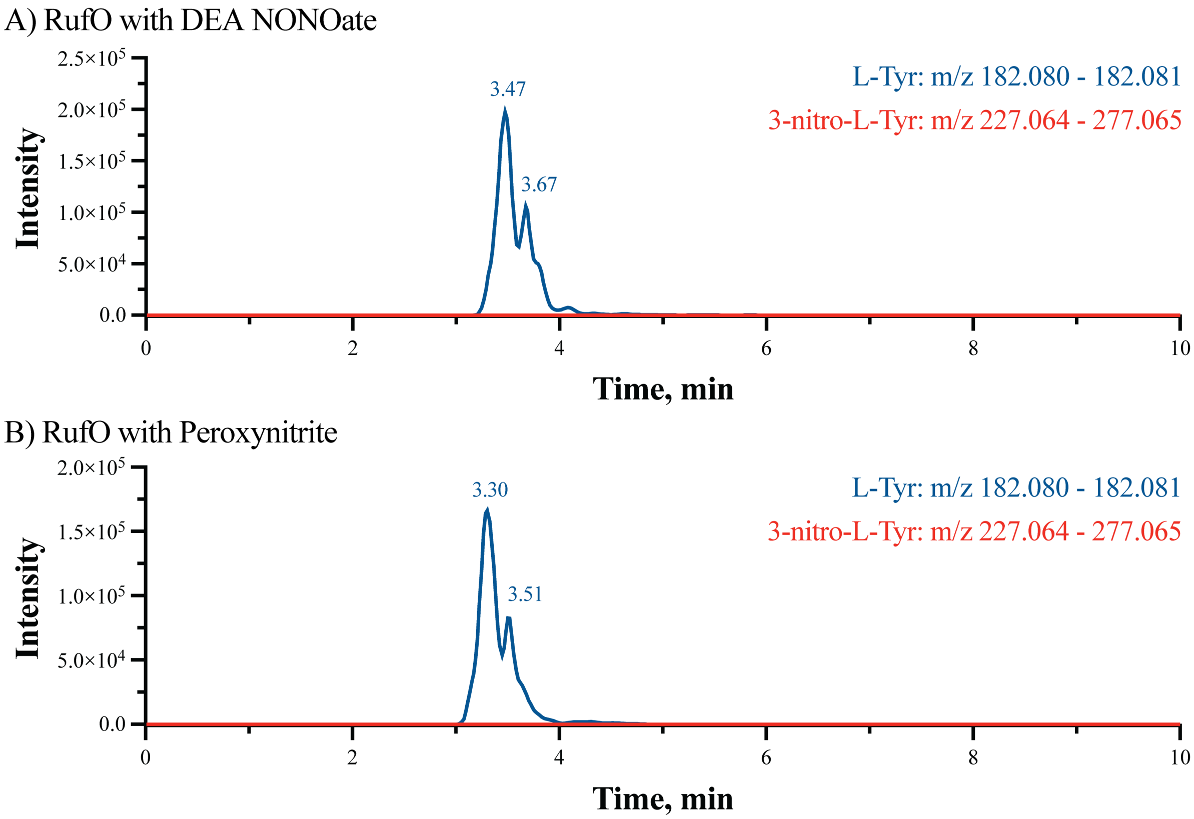

Figure S6. LC-MS analysis of single turnover RufO reactions with excess enzyme. EICs monitoring the presence of L-Tyr and 3-nitro-L-Tyr are shown, where either (A) DEA NONOate or (B) peroxynitrite were included in the RufO reaction mixture. No detectable product was produced by RufO in either condition. The enzymatic mixture was prepared under anaerobic conditions with 150 µM RufO, 1 mM L-Tyr, and 1 mM DEA NONOate or peroxynitrite in 25 mM Tris-Cl, pH 8.0, and 5% glycerol.

A) RufO with DEA NONOate

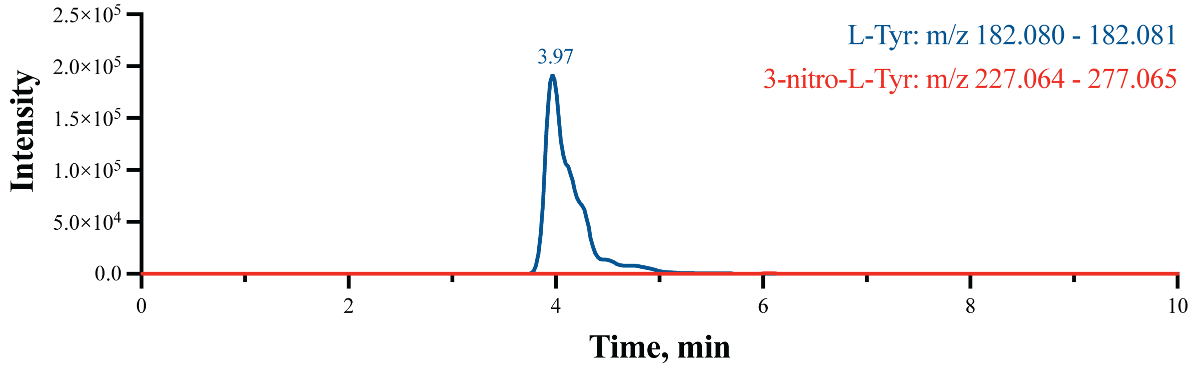

B) RufO with Peroxynitrite

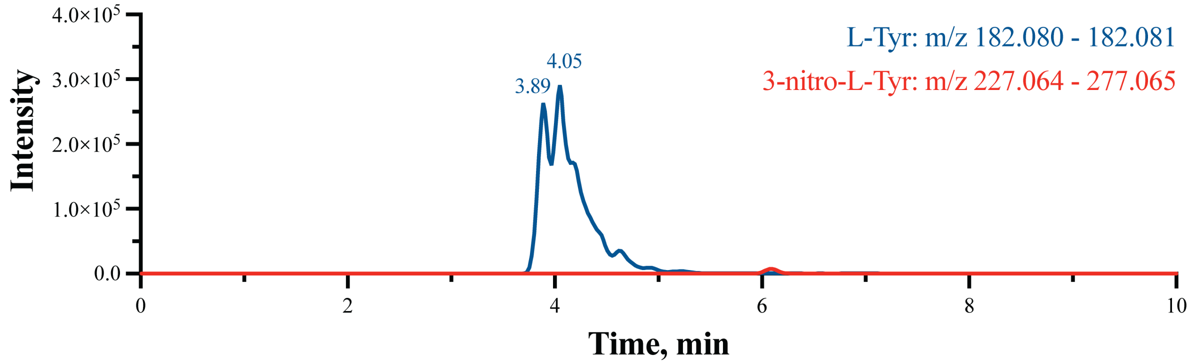

Figure S7. LC-MS analysis of RufO activity with ferredoxin and ferredoxin-NADP$^+$ reductase. EICs monitoring the presence of L-Tyr and 3-nitro-L-Tyr are shown, where either (A) DEA NONOate or (B) peroxynitrite were included in the RufO reaction mixture. The enzymatic mixture was prepared under aerobic conditions with 4 µM RufO, 1 mM L-Tyr, 1 mM DEA NONOate or peroxynitrite, 1 mM NADPH, 0.01 mg/mL ferredoxin, and 0.1 U/mL ferredoxin-NADP$^+$ reductase in 25 mM Tris-Cl, pH 8.0, and 5% glycerol.

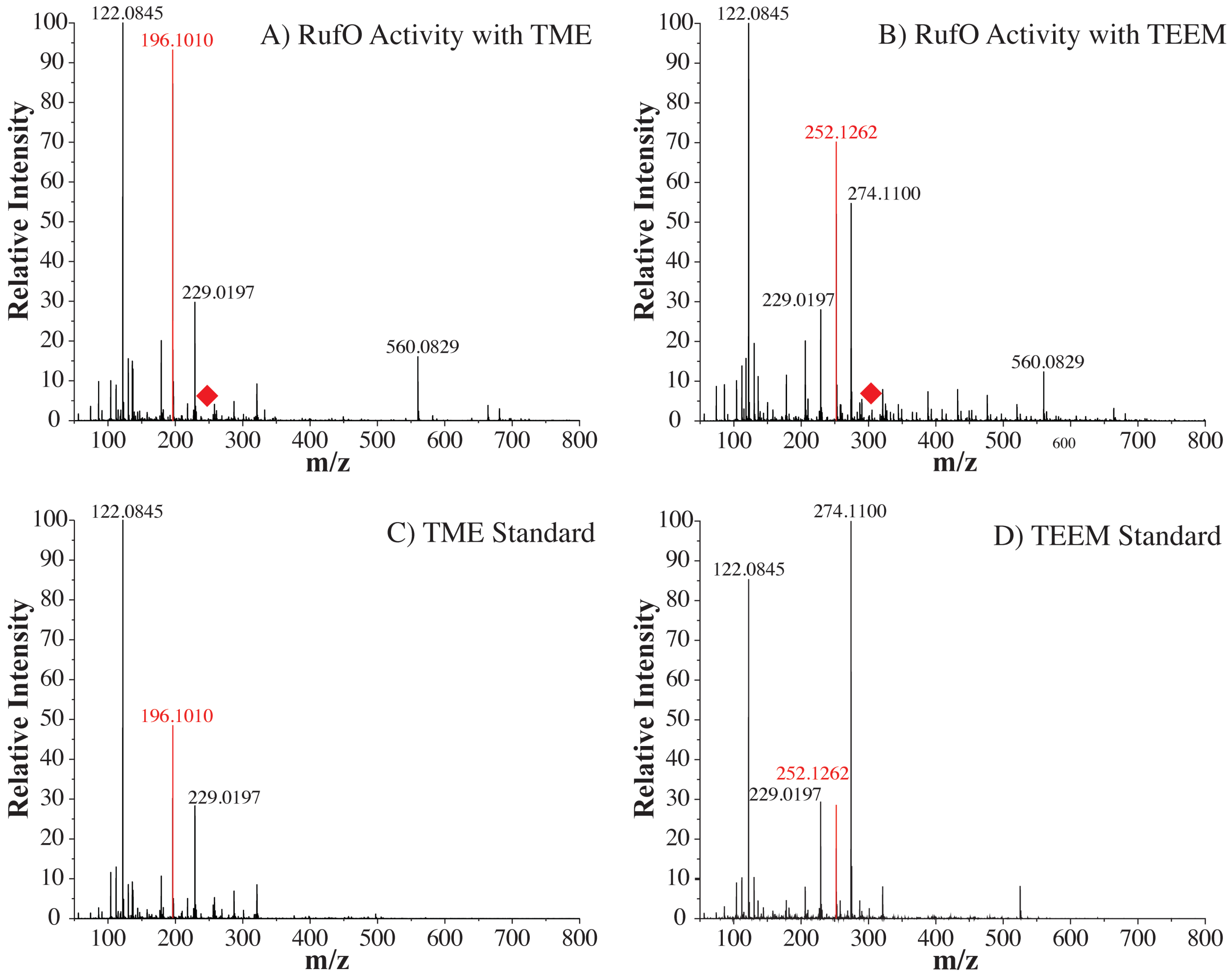

Figure S8. Direct injection MS analysis of single turnover RufO reactions with TME or TEEM. Full mass spectra from direct injection of reaction mixtures with (A) TME and (B) TEEM, as well as (C) a 1 mM TME standard and (D) a 1 mM TEEM standard. Red peaks highlight either TME (calculated m/z [m+H$^+$] = 196.090) or TEEM (calculated m/z [m+H$^+$] = 252.116), while the red diamond indictes where product formation is expected. The enzymatic mixtures were prepared under anaerobic conditions with 4 µM RufO, 1 mM TME or TEEM, 1 mM NADH, and 1 mM DEA NONOate in 25 mM Tris-Cl, pH 8.0, and 5% glycerol. Small molecule standards were prepared in 25 mM Tris-Cl, pH 8.0, and 5% glycerol. No m/z peaks corresponding to nitrated TME (calculated m/z [m+H$^+$] = 242.082) or TEEM (calculated m/z [m+H$^+$] = 298.109) were detected.

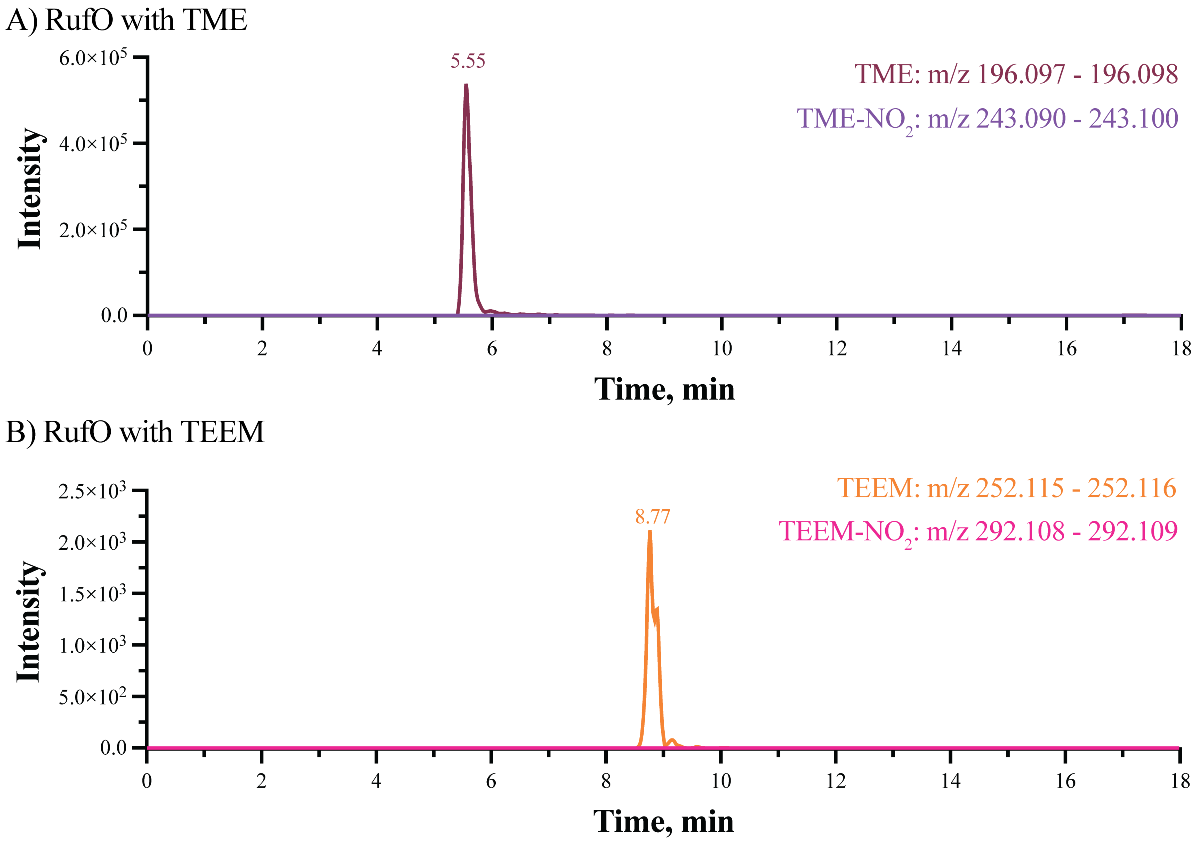

Figure S9. LC-MS analysis of single turnover RufO reactions with L-Tyr analogues and excess enzyme. EICs monitoring the presence of L-Tyr analogues and nitrated analogues are shown, where either (A) TME or (B) TEEM were included in the RufO reaction mixture. No detectable product was produced by RufO in either condition. The enzymatic mixture was prepared under anaerobic conditions with 150 µM RufO, 1 mM TME or TEEM, and 1 mM DEA NONOate in 25 mM Tris-Cl, pH 8.0, and 5% glycerol.

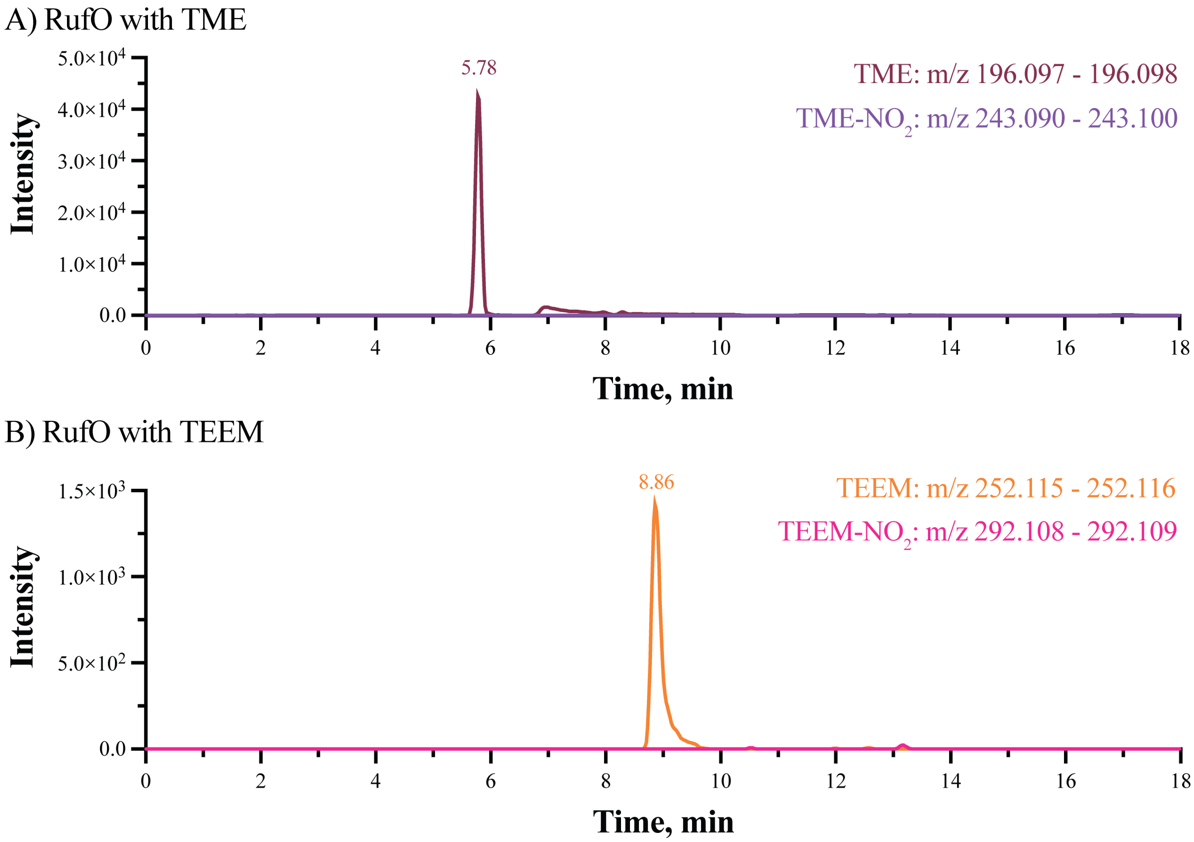

Figure S10. LC-MS analysis of RufO activity with L-Tyr analogues, ferredoxin, and ferredoxin-NADP$^+$ reductase. EICs monitoring the presence of L-Tyr analogues and nitrated analogues are shown, where either (A) TME or (B) TEEM were included in the RufO reaction mixture. No detectable product was produced by RufO in either condition. The enzymatic mixture was prepared under aerobic conditions with 4 µM RufO, 1 mM TME or TEEM, 1 mM DEA NONOate, 1 mM NADPH, 0.01 mg/mL ferredoxin, and 0.1 U/mL ferredoxin-NADP$^+$ reductase in 25 mM Tris-Cl, pH 8.0, and 5% glycerol.

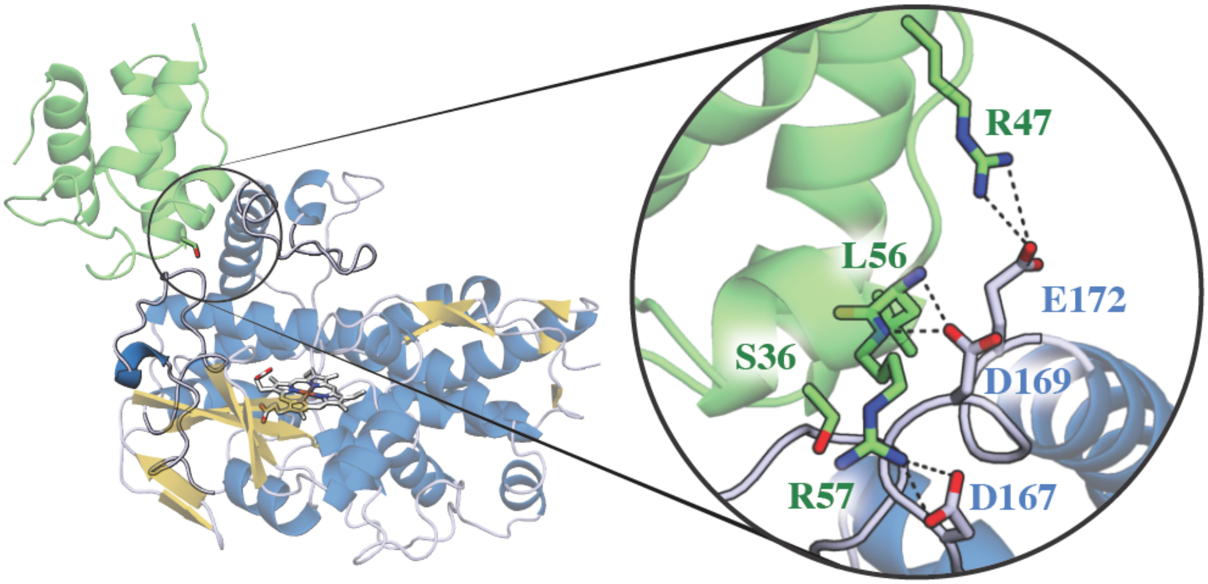

Figure S11. Interactions between the PCP-domain from module 3 of RufT and RufO. Cartoon model depicting the docked complex. Hydrogen bonding interactions between residues on the FG-loop and the PCP-domain are highlighted. The PCP-domain is colored in green and RufO is colored as in the main text.

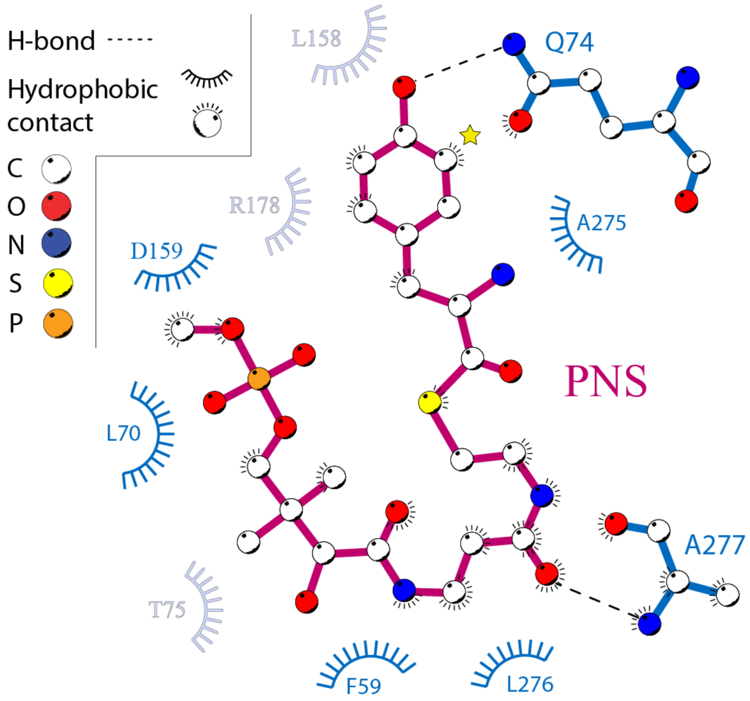

Figure S12. Interaction diagram for L-Tyr-bound phosphopantetheinyl arm (PNS) bound to active site of RufO. The carbon on L-Tyr that receives the nitro-group via direction nitration by RufO is marked with a star. Note that the substrate, heme cofactor, and RufO are colored as in the main text.

Supplementary References

1. Tomita, H.; Katsuyama, Y.; Minami, H.; Ohnishi, Y., Identification and characterization of a bacterial cytochrome P450 monooxygenase catalyzing the 3-nitration of tyrosine in rufomycin biosynthesis. *J. Biol. Chem.* **2017,** *292* (38), 15859-15869.
2. Inoue, H.; Nojima, H.; Okayama, H., High efficiency transformation of Escherichia coli with plasmids. *Gene* **1990,** *96* (1), 23-28.
3. Gasteiger, E.; Hoogland, C.; Gattiker, A.; Duvaud, S. e.; Wilkins, M. R.; Appel, R. D.; Bairoch, A., Protein identification and analysis tools on the ExPASy server. In *The Proteomics Protocols Handbook*, Walker, J. M., Ed. Humana Press: Totowa, NJ, 2005; pp 571-607.
4. Kabsch, W., XDS. *Acta Crystallogr. D* **2010,** *66* (2), 125-132.
5. Evans, P. R.; Murshudov, G. N., How good are my data and what is the resolution? *Acta Crystallogr. D* **2013,** *69* (7), 1204-1214.
6. Adams, P. D.; Afonine, P. V.; Bunkoczi, G.; Chen, V. B.; Davis, I. W.; Echols, N.; et al., PHENIX: a comprehensive python-based system for macromolecular structure solution. *Acta Crystallogr. D* **2010,** *66* (2), 213-221.
7. Emsley, P.; Cowtan, K., Coot: model-building tools for molecular graphics. *Acta Crystallogr. D* **2004,** *60* (12 Part 1), 2126-2132.
8. Emsley, P.; Lohkamp, B.; Scott, W. G.; Cowtan, K., Features and development of Coot. *Acta Crystallogr. D* **2010,** *66* (4), 486-501.
9. Williams, C. J.; Headd, J. J.; Moriarty, N. W.; Prisant, M. G.; Videau, L. L.; Deis, L. N.; et al., MolProbity: more and better reference data for improved all-atom structure validation. *Protein Sci.* **2018,** *27* (1), 293-315.
10. Joosten, R. P.; Long, F.; Murshudov, G. N.; Perrakis, A., The PDB_REDO server for macromolecular structure model optimization. *IUCrJ* **2014,** *1* (4), 213-220.
11. Schrodinger, LLC, The PyMOL Molecular Graphics System, Version 1.8. 2015.
12. Tian, W.; Chen, C.; Lei, X.; Zhao, J.; Liang, J., CASTp 3.0: computed atlas of surface topography of proteins. *Nucleic Acids Res.* **2018,** *46* (W1), W363-W367.
13. Panjikar, S.; Parthasarathy, V.; Lamzin, V. S.; Weiss, M. S.; Tucker, P. A., Auto-Rickshaw: an automated crystal structure determination platform as an efficient tool for the validation of an X-ray diffraction experiment. *Acta Crystallogr. D* **2005,** *61* (4), 449-457.
14. Panjikar, S.; Parthasarathy, V.; Lamzin, V. S.; Weiss, M. S.; Tucker, P. A., On the combination of molecular replacement and single-wavelength anomalous diffraction phasing for automated structure determination. *Acta Crystallogr. D* **2009,** *65* (10), 1089-1097.
15. Jumper, J.; Evans, R.; Pritzel, A.; Green, T.; Figurnov, M.; Ronneberger, O.; et al., Highly accurate protein structure prediction with AlphaFold. *Nature* **2021,** *596* (7873), 583-589.
16. Mirdita, M.; Schütze, K.; Moriwaki, Y.; Heo, L.; Ovchinnikov, S.; Steinegger, M., ColabFold: making protein folding accessible to all. *Nat. Methods* **2022,** *19* (6), 679-682.
17. Taylor, G., Introduction to phasing. *Acta Crystallogr. D* **2010,** *66* (4), 325-338.
18. Doran, P. M., Chapter 10 - Mass transfer. In *Bioprocess Engineering Principles, 2nd ed.*, Doran, P. M., Ed. Academic Press: London, 2013; pp 379-444.
19. Prieto, C.; García-Estrada, C.; Lorenzana, D.; Martín, J. F., NRPSsp: non-ribosomal peptide synthase substrate predictor. *Bioinformatics* **2011,** *28* (3), 426-427.
20. Bachmann, B. O.; Ravel, J., Chapter 8 Methods for in silico prediction of microbial polyketide and nonribosomal peptide biosynthetic pathways from DNA sequence data. In *Meth. Enzymol.*, Academic Press: 2009; Vol. 458, pp 181-217.
21. Waterhouse, A. M.; Procter, J. B.; Martin, D. M. A.; Clamp, M.; Barton, G. J., Jalview version 2—a multiple sequence alignment editor and analysis workbench. *Bioinformatics* **2009,** *25* (9), 1189-1191.
22. Dominguez, C.; Boelens, R.; Bonvin, A. M. J. J., HADDOCK: A protein−protein docking approach based on biochemical or biophysical information. *J. Amer. Chem. Soc* **2003,** *125* (7), 1731-1737.

23. van Zundert, G. C. P.; Rodrigues, J. P. G. L. M.; Trellet, M.; Schmitz, C.; Kastritis, P. L.; Karaca, E.; et al., The HADDOCK2.2 web server: user-friendly integrative modeling of biomolecular complexes. *J. Mol. Biol.* **2016,** *428* (4), 720-725.
24. Case, D.; Belfon, K.; Ben-Shalom, I.; Brozell, S.; Cerutti, D.; Cheatham, T.; III, V. C.; Darden, T.; Duke, R.; Giambasu, G., AMBER2020, University of California, San Fransisco. *J. Amer. Chem. Soc.* **2020,** *142*, 3823-3835.
25. Jakalian, A.; Jack, D. B.; Bayly, C. I., Fast, efficient generation of high-quality atomic charges. AM1-BCC model: II. Parameterization and validation. *J. Comput. Chem.* **2002,** *23* (16), 1623-1641.
26. Anandakrishnan, R.; Aguilar, B.; Onufriev, A. V., H++ 3.0: automating pK prediction and the preparation of biomolecular structures for atomistic molecular modeling and simulations. *Nucleic Acids Res.* **2012,** *40* (W1), W537-W541.
27. Gordon, J. C.; Myers, J. B.; Folta, T.; Shoja, V.; Heath, L. S.; Onufriev, A., H++: a server for estimating p K as and adding missing hydrogens to macromolecules. *Nucleic Acids Res.* **2005,** *33* (suppl_2), W368-W371.
28. Myers, J.; Grothaus, G.; Narayanan, S.; Onufriev, A., A simple clustering algorithm can be accurate enough for use in calculations of pKs in macromolecules. *Proteins: Struct. Funct. Bioinforma.* **2006,** *63* (4), 928-938.
29. Bayly, C. I.; Cieplak, P.; Cornell, W.; Kollman, P. A., A well-behaved electrostatic potential based method using charge restraints for deriving atomic charges: the RESP model. *J. Phys. Chem.* **1993,** *97* (40), 10269-10280.
30. Li, P.; Merz, K. M., Jr., MCPB.py: a python based metal center parameter builder. *J. Chem. Inf. Model.* **2016,** *56* (4), 599-604.
31. Becke, A., Density-functional thermochemistry. III. The role of exact exchange. *J. Chem. Phys.* **1993,** *98*, 5648-5652.
32. Lee, C.; Yang, W.; Parr, R. G., Development of the Colle-Salvetti correlation-energy formula into a functional of the electron density. *Phys. Rev. B* **1988,** *37* (2), 785-789.
33. Frisch, M. J. T., G. W.; Schlegel, H. B.; Scuseria, G. E.; Robb, M. A.; Cheeseman, J. R.; Scalmani, G.; et al., Gaussian 09, revision E.01. **2009**, 20-44.
34. Lindorff-Larsen, K.; Piana, S.; Palmo, K.; Maragakis, P.; Klepeis, J. L.; Dror, R. O.; Shaw, D. E., Improved side-chain torsion potentials for the Amber ff99SB protein force field. *Proteins Struct. Funct. Bioinforma.* **2010,** *78* (8), 1950-1958.
35. Wang, J.; Cieplak, P.; Kollman, P. A., How well does a restrained electrostatic potential (RESP) model perform in calculating conformational energies of organic and biological molecules? *J. Comput. Chem.* **2000,** *21* (12), 1049-1074.
36. Sousa da Silva, A. W.; Vranken, W. F., ACPYPE - AnteChamber PYthon Parser interfacE. *BMC Res. Notes* **2012,** *5* (1), 367.
37. Abraham, M.; van der Spoel, D.; Lindahl, E.; Hess, B., The GROMACS Development Team, GROMACS User Manual 2019.
38. Bussi, G.; Donadio, D.; Parrinello, M., Canonical sampling through velocity rescaling. *J. Chem. Phys.* **2007,** *126* (1), 014101.
39. Parrinello, M.; Rahman, A., Polymorphic transitions in single crystals: a new molecular dynamics method. *J. Appl. Phys.* **1981,** *52* (12), 7182-7190.
40. Hess, B.; Bekker, H.; Berendsen, H. J. C.; Fraaije, J. G. E. M., LINCS: a linear constratint solver for molecular simulations. *J. Comput. Chem.* **1997,** *18* (12), 1463-1472.
41. Pettersen, E. F.; Goddard, T. D.; Huang, C. C.; Couch, G. S.; Greenblatt, D. M.; Meng, E. C.; Ferrin, T. E., UCSF Chimera—a visualization system for exploratory research and analysis. *J. Comput. Chem.* **2004,** *25* (13), 1605-1612.
42. Louka, S.; Barry, S. M.; Heyes, D. J.; Mubarak, M. Q. E.; Ali, H. S.; Alkhalaf, L. M.; et al., Catalytic mechanism of aromatic nitration by cytochrome P450 TxtE: involvement of a ferric-peroxynitrite intermediate. *J. Amer. Chem. Soc* **2020,** *142* (37), 15764-15779.
